# Lessons from Hepatocyte-Specific *Cyp51* Knockout Mice: Impaired Cholesterol Synthesis Leads to Oval Cell-Driven Liver Injury

**DOI:** 10.1038/srep08777

**Published:** 2015-03-05

**Authors:** Gregor Lorbek, Martina Perše, Jera Jeruc, Peter Juvan, Francisco M. Gutierrez-Mariscal, Monika Lewinska, Rolf Gebhardt, Rok Keber, Simon Horvat, Ingemar Björkhem, Damjana Rozman

**Affiliations:** 1 Centre for Functional Genomics and Bio-Chips, Institute of Biochemistry, Faculty of Medicine, University of Ljubljana, Ljubljana, Slovenia; 2 Medical Experimental Centre, Institute of Pathology, Faculty of Medicine, University of Ljubljana, Ljubljana, Slovenia; 3 Institute of Pathology, Faculty of Medicine, University of Ljubljana, Ljubljana, Slovenia; 4 Institute of Biochemistry, Faculty of Medicine, University of Leipzig, Leipzig, Germany; 5 Department of Animal Science, Biotechnical Faculty, University of Ljubljana, Ljubljana, Slovenia; 6 National Institute of Chemistry, Ljubljana, Slovenia; 7 Department of Laboratory Medicine, Division of Clinical Chemistry, Karolinska Institute, Karolinska University Hospital, Huddinge, Sweden

## Abstract

We demonstrate unequivocally that defective cholesterol synthesis is an independent determinant of liver inflammation and fibrosis. We prepared a mouse hepatocyte-specific knockout (LKO) of lanosterol 14α-demethylase (CYP51) from the part of cholesterol synthesis that is already committed to cholesterol. LKO mice developed hepatomegaly with oval cell proliferation, fibrosis and inflammation, but without steatosis. The key trigger was reduced cholesterol esters that provoked cell cycle arrest, senescence-associated secretory phenotype and ultimately the oval cell response, while elevated CYP51 substrates promoted the integrated stress response. In spite of the oval cell-driven fibrosis being histologically similar in both sexes, data indicates a female-biased down-regulation of primary metabolism pathways and a stronger immune response in males. Liver injury was ameliorated by dietary fats predominantly in females, whereas dietary cholesterol rectified fibrosis in both sexes. Our data place defective cholesterol synthesis as a focus of sex-dependent liver pathologies.

Cholesterol is a versatile molecule that serves as a major constituent of cell membranes, a precursor of bile acids (BA) and steroid hormones, an inducer of the Hedgehog family of morphogens, and a regulator of the cell cycle^1^,^2^. Various aspects of cholesterol homeostasis, such as intestinal absorption^3^, blood transport^4^, and cellular trafficking^5^ are extensively studied in the pathogenesis of atherosclerosis, the leading cause of mortality in the developed world. Given that cardiovascular disease is tightly linked to the metabolic syndrome, where non-alcoholic fatty liver disease (NAFLD) has been recognized as its hepatic manifestation^6^, deranged hepatic cholesterol synthesis might have broad pathogenic implications. Namely, recent data associate increased hepatic cholesterol synthesis with NAFLD^7^ and de-regulated hepatic synthesis with its severity^8^. Mice lacking a two-channel pore 2 that is involved in intracellular trafficking of LDL cholesterol are highly susceptible to hepatic cholesterol overload and liver damage consistent with NAFLD^9^.

On the other side of the cholesterol-associated disease spectrum are the striking examples of cholesterol deficiency. Inborn errors of cholesterol synthesis are frequently lethal^10^. When compatible with life, they manifest in mental retardation and multiple congenital defects^11^, probably due to the improper activation of the Hedgehog signaling pathway and/or accumulation of potentially toxic cholesterol intermediates^10^. Progressive cholestasis and liver fibrosis were reported in up to 16% of Smith-Lemli-Opitz syndrome patients^11^, indicating that metabolic causes of liver injury might be extended also to cholesterol.

The fact that the full knockout (KO) mouse models of cholesterogenic genes are embryonic or perinatal lethal certainly represents an obstacle for follow-up studies^10^. The hepatocyte-specific KO of 3-hydroxy-3-methylglutaryl-Coenzyme A reductase (HMGCR) that causes steatosis with jaundice, hypoglycemia and eventually death, does not prove that the observed pathologies are due to the absence of cholesterol, since the isoprenoid, ubiquinone and heme A pathways are also depleted^12^. Cholesterol is a precursor of oxysterols that are crucial hepatic signaling molecules working through the liver X receptor (LXR)^13^. Cholesterol is converted also to BAs that activate farnesoid X receptor (FXR) and the G protein-coupled receptor TGR5, further affecting metabolism, together with inflammation, fibrosis and carcinogenesis^14^. It is thus crucial to determine the role of hepatocyte cholesterol synthesis in the liver by leaving the isoprenoid pathway intact.

We focused on lanosterol 14α-demethylase (CYP51) from the late part of the cholesterol synthesis pathway that is already committed to cholesterol^15^. Due to embryonic lethality of the full *Cyp51* KO^16^, we inactivated the gene specifically in hepatocytes. Liver is a sexually dimorphic organ with crucial metabolic pathways differing between females and males^17^,^18^. It is thus interesting to question whether cholesterol synthesis disharmony is responsible for sex dependent liver pathophysiology.

## Results

### Hepatocyte Loss of *Cyp51* Causes Pleiotropic Body Effects with Hepatomegaly, Oval cell Response (Ductular Reaction), Inflammation and Fibrosis

**Figure 1:**
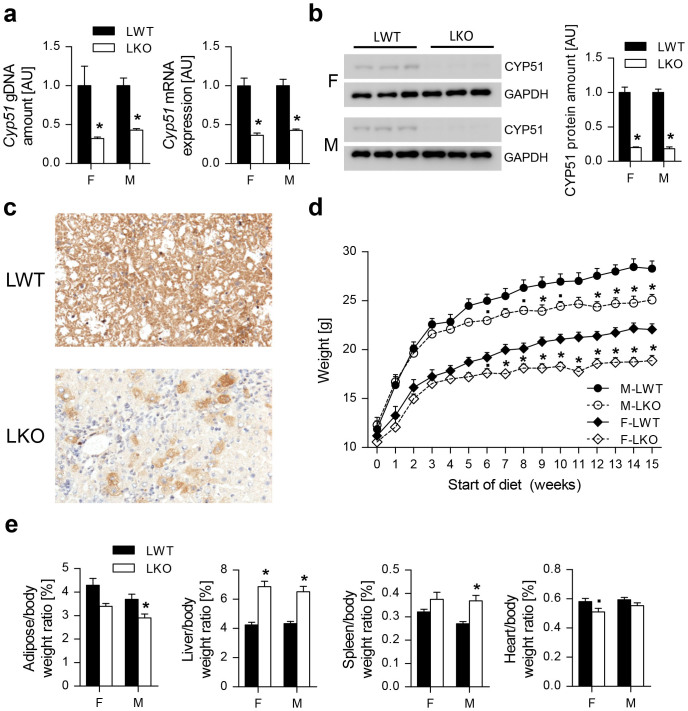
Hepatic loss of *Cyp51* causes pleiotropic body effects with hepatomegaly. (a) qPCR determination of *Cyp51* gDNA and mRNA of the LWT and LKO mice of both sexes (n = 5) in the liver. (b) Western blot analysis of hepatic CYP51 (n = 3) and a corresponding relative quantification graph. GAPDH was used as a loading control. (c) Representative immunohistochemistry of CYP51 in the liver. Original magnification 200×. (d) Growth curves of the female and male LWT and LKO mice on the standard low-fat no-cholesterol (LFnC) diet (n = 9–13). (e) Various organ to body weight ratios of the mice on the LFnC diet (n = 9–13). Columns represent means and error bars represent SEMs. Uncropped western blot is presented in Supplementary Figure 1a. AU – arbitrary units. * p < 0.05;**·** p < 0.1.

The hepatocyte-specific *Cyp51* KO mice (*Cyp51^flox^*^/*flox*^; *Alb-Cre* or LKO) of both sexes were born indistinguishable from their control littermates lacking the *Cre* transgene (*Cyp51^flox^*^/*flox*^ or LWT). To ascertain the efficiency and liver-specificity of *Cyp51* excision, we quantified the remaining *Cyp51* gDNA, mRNA and proteins in livers and kidneys. About 40% of *Cyp51* gDNA (exons 3 and 4) remained in the livers of LKO mice. This led to a roughly 60% decrease of mRNA and 80% decrease of the CYP51 protein (Fig. 1a, b). No excision was observed in the kidneys, confirming the specificity of *Cyp51* deletion in hepatocytes (Supplementary Fig. 1b, c). CYP51 immunohistochemistry (Fig. 1c) showed singular or small foci of stained periportal hepatocytes that potentially originated from the oval cell compartment as was also demonstrated by others^19^.

LKO mice fed standard laboratory chow diet (low-fat no-cholesterol diet or LFnC) gained less weight after 5–7 weeks (statistically significant reduction of adipose mass in males), but otherwise developed normally (Fig. 1d, e). The most prominent feature was a 50% increase in the liver/body weight ratio. Other significant features were an increased spleen/body weight ratio in males and reduced heart/body weight ratio in females, whereas the kidneys were not affected (Fig. 1e, Supplementary Fig. 1d). Therefore we focused mainly on the liver.

**Figure 2:**
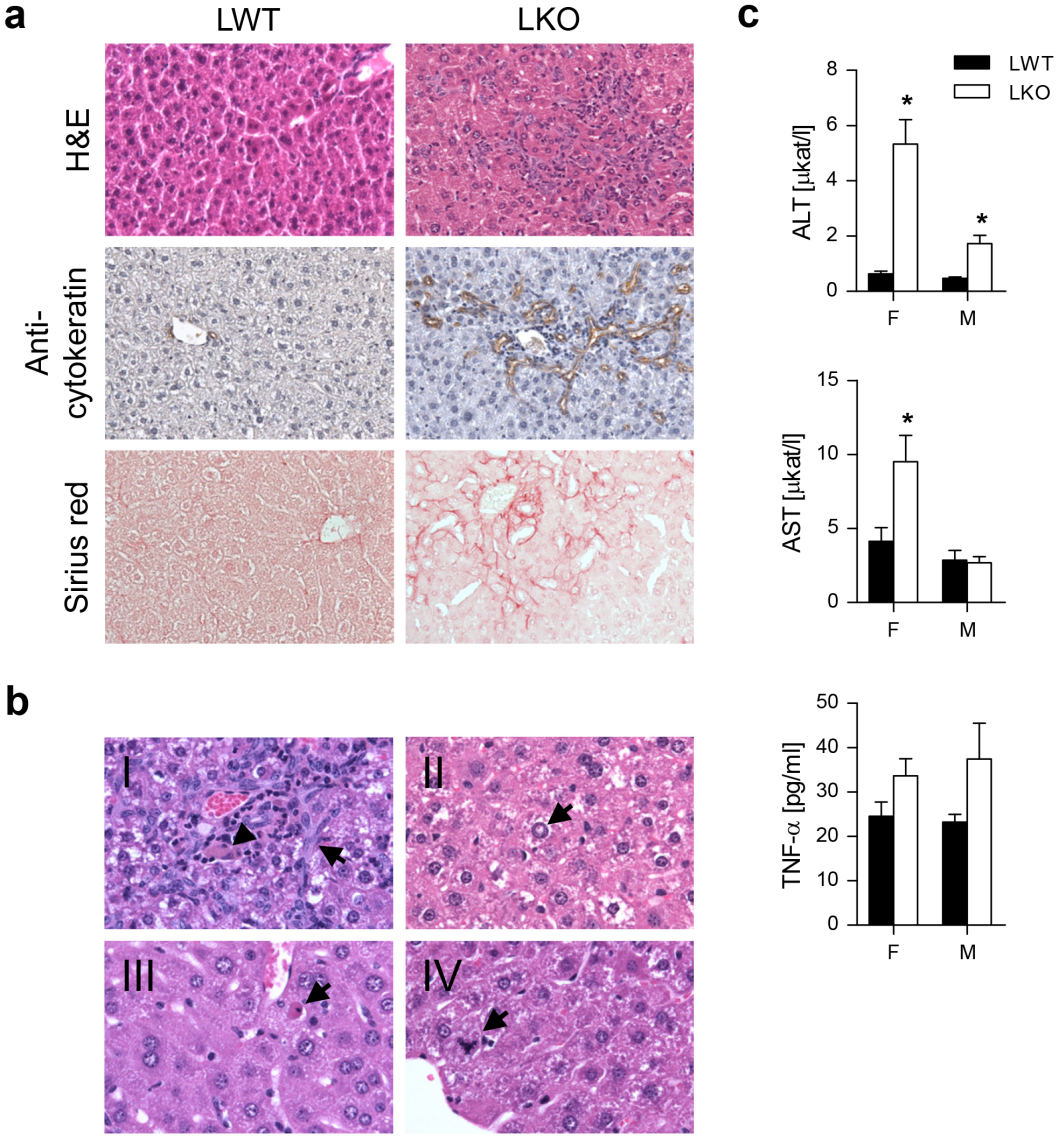
Hepatocyte-specific deletion of *Cyp51* induces hepatic ductular reaction with inflammation and fibrosis. (a) Representative hematoxylin and eosin staining, immunohistochemistry for pan-cytokeratins and Sirius red staining of the LWT and LKO mice liver sections. Original magnification 200×. (b) The observed liver pathologies on the hematoxylin and eosin-stained sections ranged from (*I*) bile duct proliferation (arrow) and grey-brown pigment indicative of cholestasis (arrow head), (*II*) nuclear vacuolation, (*III*) singular apoptotic and (*IV*) mitotic hepatocytes. Original magnification 400×. (c) Plasma levels of alanine aminotransferase (ALT), aspartate aminotransferase (AST) (n = 7–8) and TNF-α (n = 4). Columns represent means and error bars represent SEMs. * p < 0.05.

A prominent oval cell proliferation (also known as the ductular reaction) was observed in LKOs of both sexes, where the severity correlated with inflammation (mononuclear and granular inflammatory cells) and fibrosis (Fig. 2a, Supplementary Fig. 2a). Fibrosis started from the portal zone, extended to the middle zone and finally bridged portal fields in a chicken-wire pattern. The latter two patterns were observed most frequently. The majority of LKOs showed increased apoptotic and mitotic activity together with hepatic nuclear vacuolization. Cholestasis was scarce and lipid infiltration (steatosis) was not detected (Fig. 2b, Supplementary Fig. 2b, Supplementary Table 1). Female LKOs had significantly elevated plasma alanine aminotransferase (ALT) and aspartate aminotransferase (AST) levels, whereas in the males only ALT was elevated. Tumor necrosis factor α (TNF-α) remained unchanged (Fig. 2c).

### Compensatory Changes in Cholesterol Homeostasis due to Ablation of *Cyp51*

**Figure 3:**
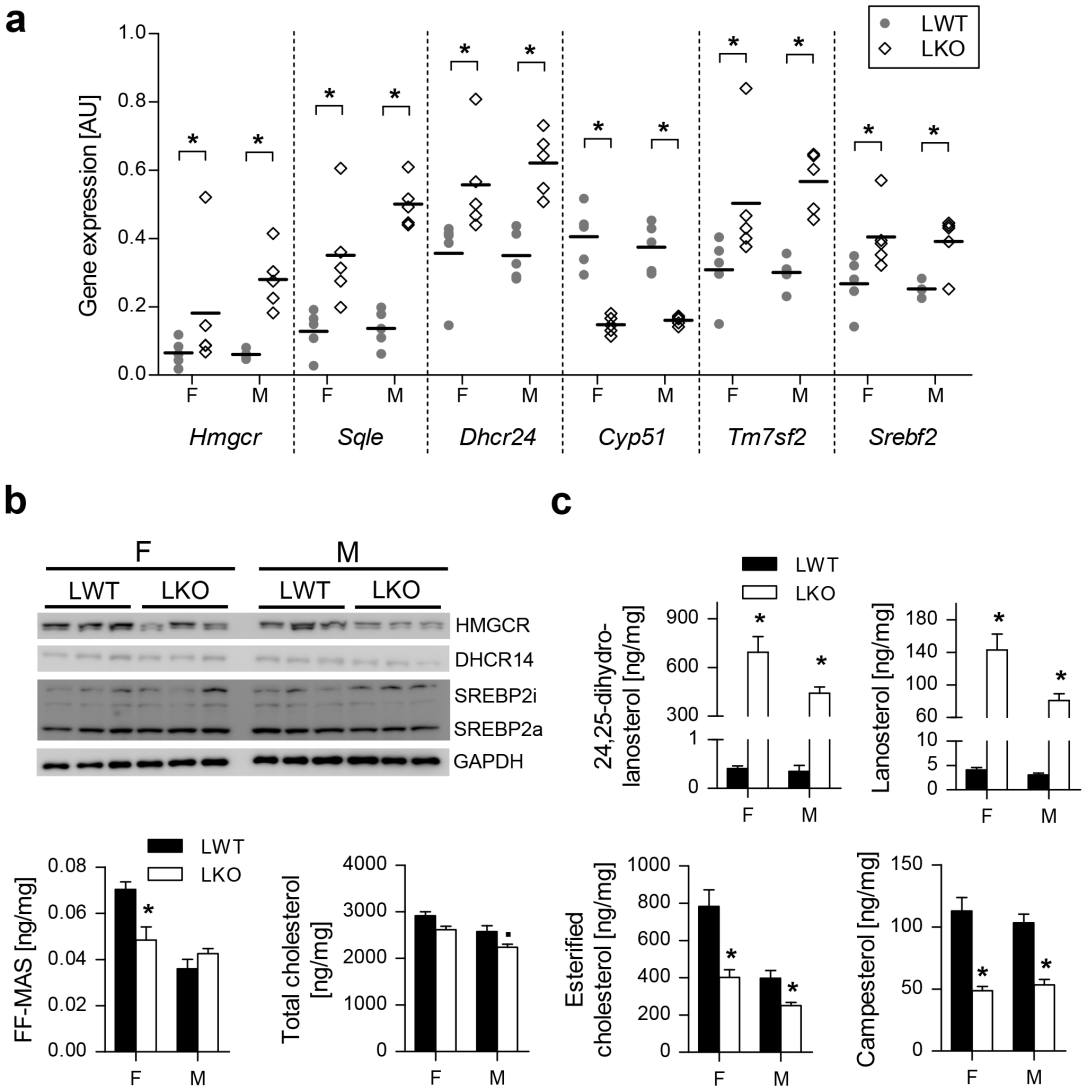
Compensatory changes in cholesterol homeostasis of LKOs lead to hepatic accumulation of CYP51 substrates and reduced cholesterol esters. (a) Dot plot showing hepatic expression of key cholesterogenic genes (n = 5). (b) Western blot analysis of proteins from the hepatic cholesterol sensing mechanisms. A representative western blot of GAPDH is presented. (c) Hepatic levels of sterols and campesterol (n = 8–9). Columns represent means and error bars represent SEMs. Uncropped western blots are presented in Supplementary Figure 10. AU – arbitrary units; SREBP2i – inactive membrane-bound form; SREBP2a – active nuclear form. * p < 0.05;**·** p < 0.1.

The block of the hepatocyte CYP51 reaction resulted in multiple compensatory changes of cholesterol synthesis at the mRNA, protein and metabolite levels. The hepatic mRNAs of key cholesterogenic genes were up-regulated (*Hmgcr, Sqle, Dhcr24, Tm7sf2*), together with *Srebf2* (Fig. 3a). Changes in the protein levels reflected post-transcriptional regulation and efforts to balance the flux through the pathway. Despite increased expression of *Hmgcr*, less HMGCR protein was produced, likely due to the buildup of lanosterol that was shown to stimulate ubiquitination and proteasomal degradation of HMGCR^20^ (Fig. 3b, c). Elevated expression of cholesterogenic genes with no significant change in active nuclear SREBP2 (SREBP2a) and inactive membrane bound SREBP2 (SREBP2i) (Fig. 3a, b) can result from the non-sterol activation of transcription^21^. Another possibility for no change in SREBP2i/a is a complex interaction between the sterol biosynthetic pathway (de-regulated sterols) and the integrated stress response (ISR)^22^: the CYP51 substrates lanosterol and 24,25-dihydrolanosterol (DHL) were substantially elevated and FF-MAS was significantly reduced in LKO females (Fig. 3c). This is surprising since the levels of DHCR14 were unchanged in both sexes (Fig. 3b).

Cholesterol homeostasis appeared to differ between the sexes: despite ablation of *Cyp51*, hepatic cholesterol was reduced only in LKO males. Interestingly, esterified cholesterol (CE) but not hepatic free cholesterol (FC) was reduced in LKOs of both sexes, together with campesterol and sitosterol that are absorbed from the intestines with cholesterol (Fig. 3c, Supplementary Fig. 3a).

**Figure 4:**
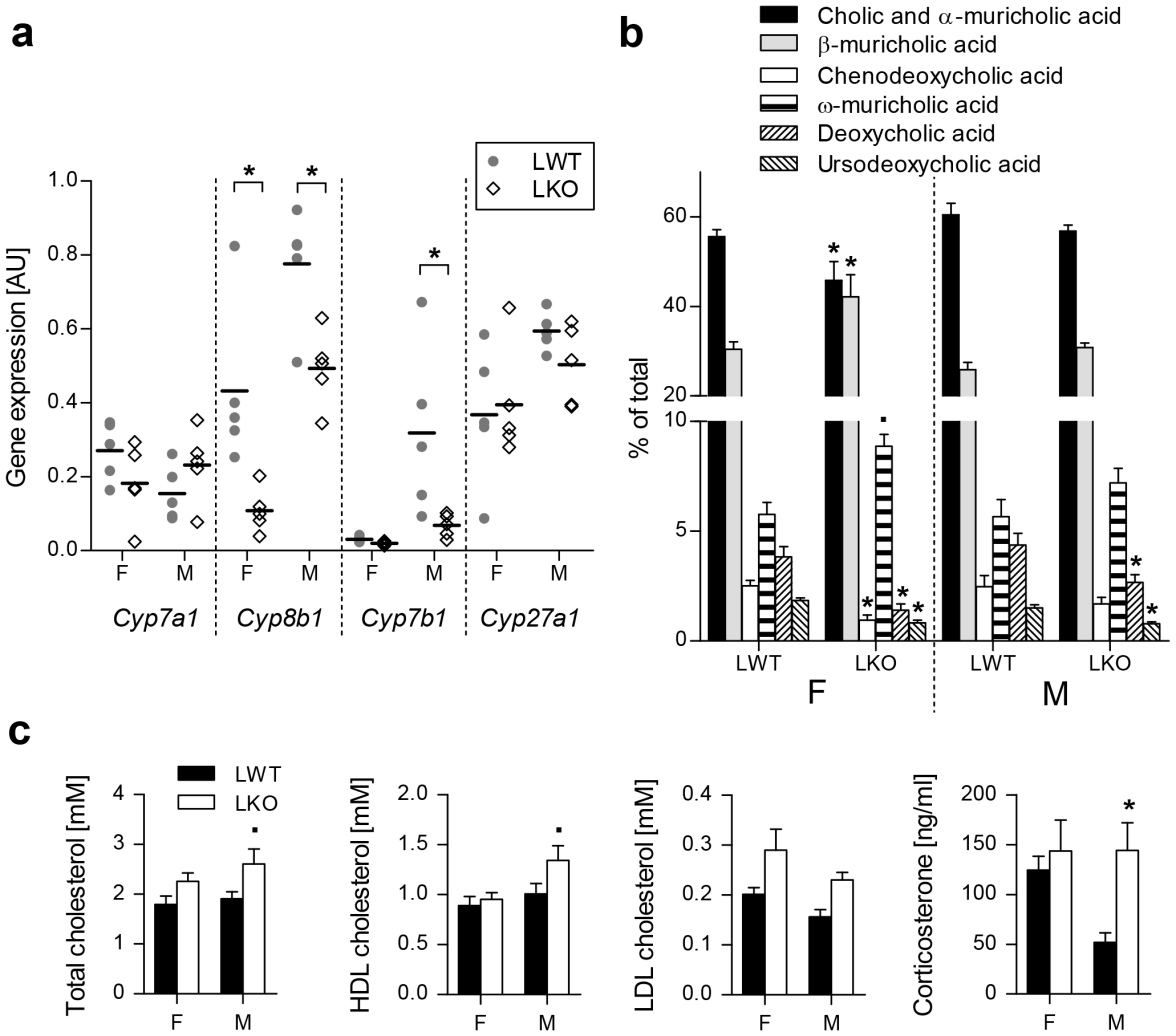
Decreased expression of bile acid synthesis genes contributes to altered gallbladder bile acid composition of LKO mice. (a) Dot plot of hepatic bile acid synthesis genes expression of the LKO and LWT mice on the LFnC diet (n = 5). (b) Relative gallbladder bile acid composition (n = 8–11) of the mice on the LFnC diet. (c) Plasma lipids profile (n = 8) and corticosterone levels (n = 5–6) of the LWTs and LKOs on the LFnC diet. Columns represent means and error bars represent SEMs. AU – arbitrary units. * p < 0.05;**·** p < 0.1.

Deregulation of hepatic sterol lipids also influenced BA homeostasis. Down-regulated *Cyp8b1* (with no change in *Cyp7a1* and *Cyp27a1*) indicated a shift in LKOs (particularly females) from cholic to chenodeoxycholic acid, which is in mice promptly converted to muricholic acids^23^ (Fig. 4a, b). Down-regulated *Cyp7b1* (Fig. 4a) together with *Hsd17b4* and *Hsd3b7* (Table 1) suggested a decrease in BA synthesis. This is in accordance with the diminished relative content of deoxycholic and ursodeoxycholic BAs that indicate a reduced enterohepatic BA circulation (Fig. 4b).

In addition to the described hepatic adaptations, the loss of hepatocyte *Cyp51* also resulted in sex-dependent adaptation of the blood lipid profile: plasma cholesterol (total and HDL) and corticosterone were significantly elevated in LKO males (Fig. 4c, Supplementary Fig. 3b).

### Gene Expression Profiling of *Cyp51* KO Livers Reveals Cell Cycle Arrest, Immune Response and Sexually Dimorphic Metabolic Adaptations

Gene expression profiling was performed to understand the global mechanisms that led to the observed liver pathologies. On the LFnC diet, 3072 differentially expressed (DE) genes were identified in male (2154 male-specific) and 2706 in female LKOs (1788 female-specific) with 918 overlapping DE genes (Supplementary Fig. 4a, b; Supplementary Tables 2, 3). Despite a substantially higher number of male-specific DE genes (majority were up-regulated; Supplementary Fig. 5a), only a handful of Kyoto Encyclopedia of Genes and Genomes (KEGG) pathways were specifically enriched in males, i.e. the terpenoid backbone synthesis or olfactory transduction (Supplementary Table 4). In females crucial metabolic pathways related to peroxisome, fatty acid and amino acid metabolism, and adipocytokine signaling were down regulated, but TGF-β1 signaling (Supplementary Fig. 5b, Supplementary Table 5) was up-regulated. From the KEGG pathways enriched in both sexes, the majority were up-regulated and linked to the immune response (B cell-, T cell-, Toll-like receptor- and chemokine signaling) or associated with apoptosis and the cell cycle. As previously indicated, BA synthesis was down-regulated (Table 1). Applying a 1.5 fold change threshold exposed DE genes with pronounced sexual dimorphism in the enriched canonical pathways (IPA™ analysis) that were in agreement with the KEGG analysis. Here the male-specific pathways mainly associated with immune response, cholesterol synthesis and HGF signaling. Male LKOs had significantly more enriched DE genes from the common immune-related pathways (i. e. the leukocyte extravasation signaling or T helper cell differentiation), again indicating a stronger activation of inflammatory pathways. The female enriched pathways were from amino acid metabolism, hepatic cholestasis, PPAR-α/RXR-α, TWEAK and Oncostatin M (Supplementary Table 6, 7).

Due to the reduction in hepatic sterols and altered BA composition we expected deregulated LXR and FXR signaling, especially since LXRα directly regulates *CYP51A1*^24^. According to the IPA analysis, the LXR/RXR pathway was enriched in both sexes, whereas FXR/RXR was borderline significant in females (Supplementary Table 6, 7). However, gene expression and protein analyses failed to show changes of LXR and its downstream targets *Abcg5/8* in LKOs (Supplementary Fig. 6).

### Dietary Fats and Cholesterol Alleviate *Cyp51* KO Liver Pathologies in Sexually Biased Manner

**Figure 5:**
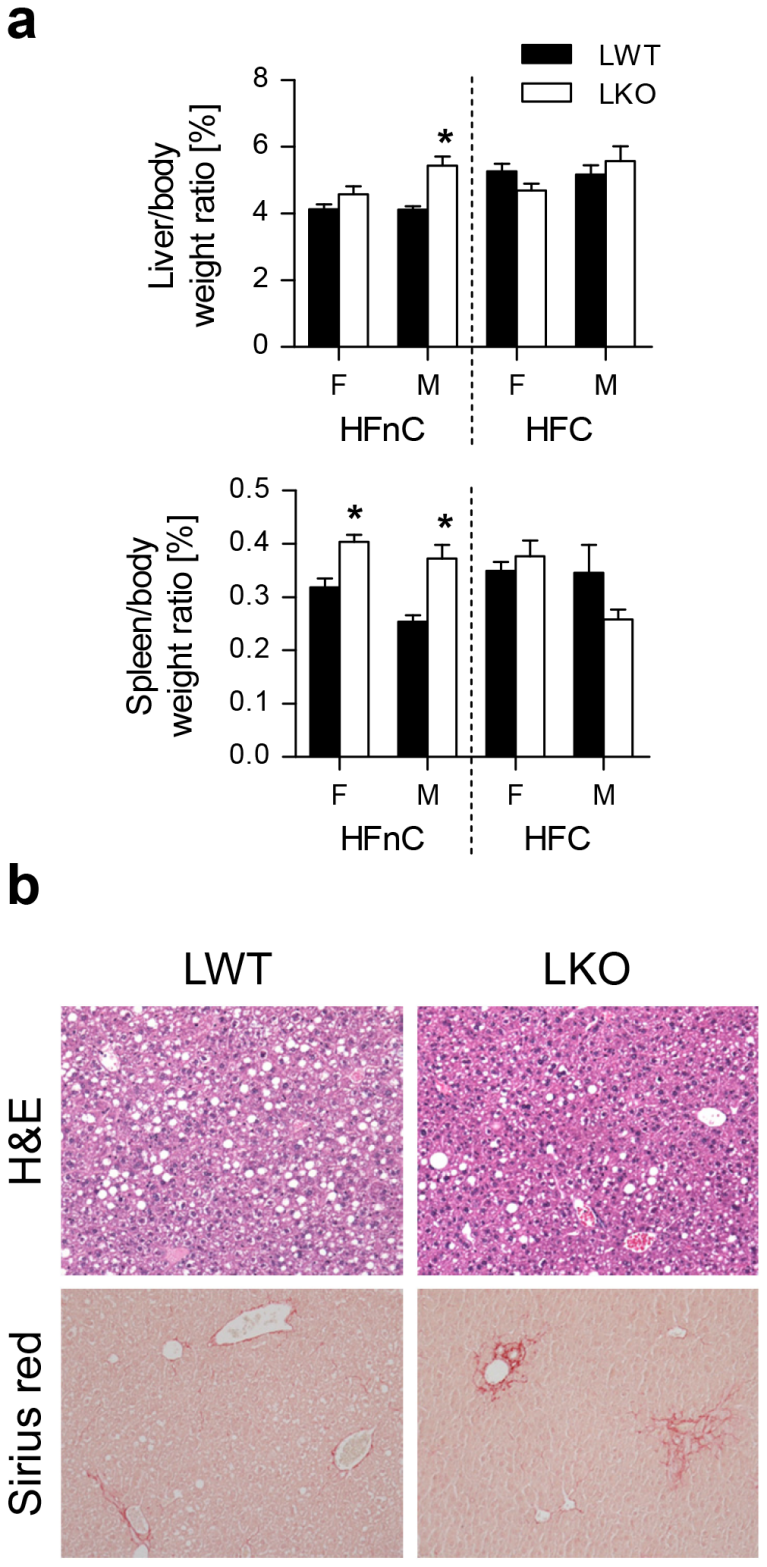
Dietary fats and cholesterol alleviate *Cyp51* KO liver pathologies in sexually biased manner. (a) Liver and spleen to body weight ratios of the LWT and LKO mice of both sexes on the high-fat diet with no (HFnC) or with cholesterol (HFC) (n = 10–13). (b) Representative photomicrographs of hematoxylin and eosin and Sirius Red staining for the LKO and LWT mice on the HFnC diet. Original magnification 100×. Columns represent means and error bars represent SEMs. * p < 0.05.

We questioned how the high-fat diet without cholesterol (HFnC) would affect the liver phenotype and whether adding a cholesterol-rich diet (high-fat diet with cholesterol or HFC) would be beneficial. The HFnC diet restored LKO growth curves, body weight and fat depots roughly to the LWT levels (Supplementary Fig. 7a, b). Hepatomegaly was no longer evident in the females, but splenomegaly appeared (Fig. 5a). On the HFC diet, LKO and LWT body parameters were indistinguishable (Fig. 5a, Supplementary Fig. 7a, b). Mice on both high-fat diets developed hepatic steatosis regardless of the genotype and sex (Fig. 5b, Supplementary Fig. 7c, Supplementary Table 1). Apart from the novel feature of steatosis, the HFC diet rescued the LKO phenotype in both sexes, while the HFnC diet improved liver pathologies predominantly in females (Supplementary Table 1). Consistently, AST and ALT were restored to normal in females on both high fat diets, while ALT remained elevated in male LKOs on the HFnC diet (Supplementary Table 8).

### Sex-Related Response of Cholesterol Homeostasis to Dietary Fats in Absence of Hepatocyte *Cyp51*

The major difference between HFnC and HFC diets is the presence of 1.25% cholesterol in the latter. In LKOs with ablated cholesterol synthesis, the HFnC diet reflects changes due to dietary fats only, while the HFC diet includes the compensatory effect of dietary cholesterol.

**Figure 6:**
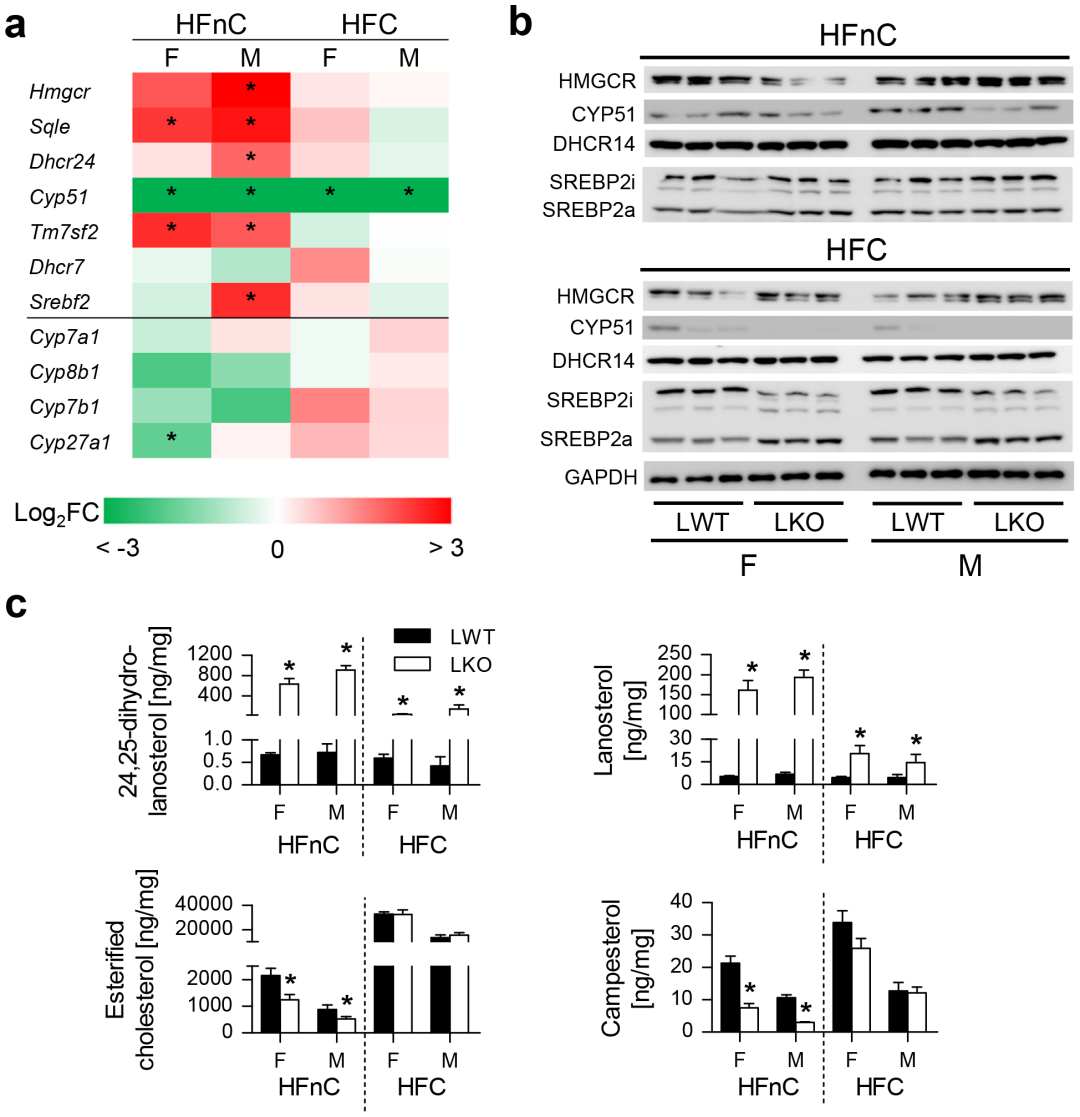
Sex-related response of cholesterol homeostasis to dietary fats in absence of hepatocyte *Cyp51*. (a) Fold change differences between the LKO and LWT mice in cholesterol homeostasis genes (n = 5) on the high-fat no-cholesterol (HFnC) and high-fat with cholesterol (HFC) diet. Statistically significant changes are marked with an asterisk (*). Color scale represents log_2_ fold change (FC) differences from −3 to 3. (b) Western blot analysis of cholesterogenic enzymes and cholesterol homeostasis proteins. A representative western blot of GAPDH is presented. (c) Hepatic levels of sterols and campesterol (n = 8–10). Columns represent means and error bars represent SEMs. Uncropped western blots are presented in Supplementary Figure 11. SREBP2i – inactive membrane-bound form; SREBP2a – active nuclear form. * p < 0.05.

In the absence of cholesterol, the up-regulation of cholesterogenic genes was less consistent in HFnC-fed female LKOs (Fig. 6a, Supplementary Table 9). Similarly as on the LFnC diet, we still observed less CYP51 protein in the knockouts, diminished HMGCR (females only), and no change in DHCR14 and SREBP2a/i (Fig. 6b). The elevated lanosterol and DHL persisted, and esterified cholesterol remained depleted (Fig. 6c, Supplementary Fig. 8a).

Deregulation of hepatic sterol lipids in a high-fat no-cholesterol environment had a modest effect on BA homeostasis. Only *Cyp27a1* from the alternative pathway of BAs was down-regulated (Fig. 6a) and the gallbladder BA composition remained unchanged apart from the reduced ursodeoxycholic acid (Supplementary Fig. 8b). Importantly, plasma cholesterol (total, LDL, HDL) concentrations were decreased only in male LKOs (Supplementary Table 8).

Next we addressed the molecular consequences of cholesterol supplementation, i.e., the HFC diet. LKO livers had to adapt to two signals: disruption of endogenous cholesterol synthesis and addition of dietary cholesterol in a high-fat environment. The gene expression in LKOs followed the (dietary) cholesterol feedback regulation (Fig. 6a, no significant changes between LWT and LKO mice), which was evident also on the protein level (barely detectable CYP51 also in LWT, no difference in DHCR14). The precursor SREBP2i was diminished in LKOs while the nuclear SREBP2a was increased (Fig. 6b). Importantly, lanosterol and DHL were elevated even in the presence of dietary cholesterol. The absorption of dietary phytosterols in LKOs reached LWT levels (Fig. 6c, Supplementary Fig. 8a) and similar compensation was observed for blood cholesterol levels (Supplementary Table 8).

The transcriptome analysis of the high-fat diet groups resulted in only 7 DE genes for male and 1 for female LKOs on the HFnC, whereas none passed the statistical criteria on the HFC diet (Supplementary Table 10). Multi-dimensional scaling revealed a clear separation of male and female mice regardless of the diet. LKO mice partitioned well from the LWTs on the LFnC diet, but the HFC-fed groups could not be distinguished according to their genotype (Supplementary Fig. 9). This was consistent with the observed histological picture.

## Discussion

The hepatocyte loss of *Cyp51* illustrates the importance of intact cholesterol synthesis for normal liver function. *Cyp51* LKOs exhibited advanced NAFLD features, such as oval cell-driven inflammation and periportal fibrosis, which are characteristic for non-alcoholic steatohepatitis (NASH) that may progress to life-threatening cirrhosis and hepatocellular carcinoma^25^. Oval cells are usually activated only when the replication of mature hepatocytes is delayed or blocked. Chronic ER and oxidative stress in NAFLD can induce hepatocyte replicative senescence that leads to a compensatory oval cell reaction^26^. It is important to note that the pathology of LKOs developed without steatosis. Steatosis was for a long time considered the primary pathogenic event in NAFLD. Increasing evidence (including data in this manuscript) suggests that steatosis and NASH are two separate conditions^27^. Our results place hepatic cholesterol synthesis as an independent determinant of liver inflammation and fibrosis.

**Figure 7:**
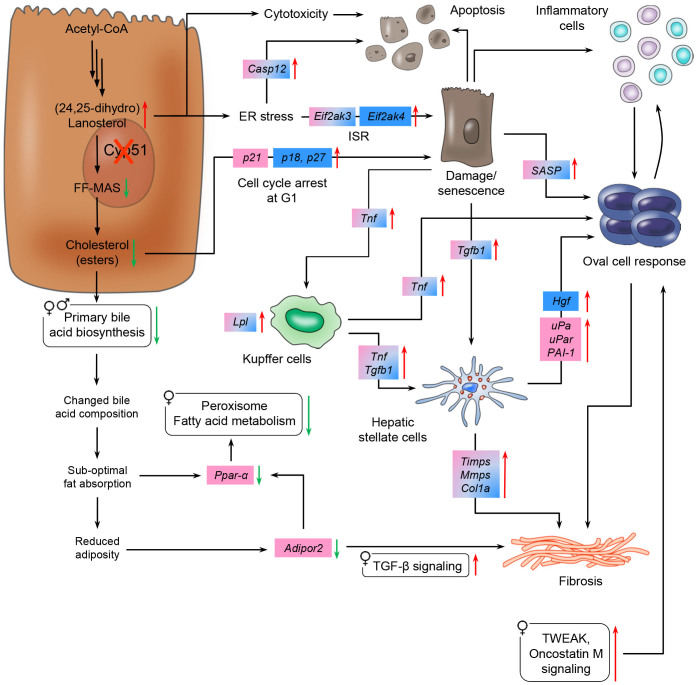
Proposed sex-specific mechanisms of LKO liver pathologies on low-fat no-cholesterol diet. Hepatic loss of *Cyp51* caused an accumulation of CYP51 substrates that resulted in hepatocyte cytotoxicity and/or endoplasmic reticulum stress. Reduced hepatic cholesterol esters led to cell cycle arrest. Resultant replicatively senescent hepatocytes secreted SASP and interacted with Kupffer cells and hepatic stellate cells that drove the oval cell response. Hepatic stellate cells and oval cell activation caused periportal fibrosis. Reduced cholesterol esters together with down-regulated bile acid biosynthesis resulted in an alteration of the bile acid composition that led to a sub-optimal fat absorption and female-specific down-regulation of *Ppar-α*. Reduced adipocytokine signaling might be connected to fibrosis progression together with increased oxidative stress *via* *Ppar-α* signaling. Blue and pink brackets represent male- and female-specific differentially expressed genes, respectively, whereas blue-pink color stands for common differentially expressed genes for both sexes. Green and red arrows indicate down- and up-regulation, respectively. Rounded rectangles represent enriched KEGG pathways in a sex-specific manner. ER, endoplasmic reticulum; ISR, integrated stress response; SASP, senescence-associated secretory phenotype.

Pathological triggers of *Cyp51* LKOs included elevated CYP51 substrates (lanosterol and DHL) and diminished cholesterol esters and BA synthesis that cumulatively decreased the hepatic lipid uptake and availability (Fig. 7). Lanosterol and DHL are normally not present in biological membranes and lanosterol supplementation fails to support the growth of cholesterol-depleted CHO-7 cells^28^. Elevated sterols possibly influenced the endoplasmic reticulum (ER) membrane structure of LKOs and provoked the ISR. This is in agreement with studies that identified small molecule activators of ISR as CYP51 inhibitors^22^. Our microarray data revealed up-regulation of multiple ISR components: *Eif2s1* in male LKOs, downstream PERK branch that mediates ER stress signaling (*Eif2ak3* in both sexes), and the GCN2 branch that signals amino acid starvation (*Eif2ak4* in males) (Fig. 7, Table 1)^22^. The latter may be more important in males, since females already have reduced amino acid catabolism. Other evidence of ER stress was the up-regulation of Caspase 12 (*Casp12*) that activates apoptotic signaling.

Lanosterol plays a role also in the post-transcriptional regulation of cholesterol synthesis, to aid in fine tuning the response to changed cholesterol availability^29^. Excess lanosterol in LKOs reduced the carbon flow through the pathway by mediating HMGCR degradation^20^.

The adaptations in cholesterol synthesis were not mediated by cholesterol-SREBP2 negative feedback loop, since nuclear SREBP2a was not significantly changed (Fig. 3b). Harding *et al*.^22^ shows that accumulation of lanosterol (due to CYP51 inhibition) leads to ISR with inhibited processing of SREBP2i to SREBP2a. Lower concentrations of CYP51 inhibitor initially elevated SREBP2a and increased expression of SREBP2 target genes. Increasing concentrations of the inhibitor gradually repressed SREBP2 activation in an ISR-dependent manner. This is in accordance with our data where increased lanosterol on the LFnC and HFnC diets was accompanied by unchanged SREBP2 (Fig. 3, Fig. 6).

The expression of the cholesterogenic genes can be increased also in the absence of increased SREBP2. For example, LXR directly silences the expression of *CYP51A1* and *FDFT1*^24^, and *Cyp51* can be activated solely by the cAMP signaling pathway^21^.

Another metabolic hallmark of *Cyp51* LKOs was diminished hepatic cholesterol esters in the absence of changes in free cholesterol (Fig. 3c, Supplementary Fig. 3a). An incremental change in FC that is not accompanied by an increase in CE is indicative of the progression from simple steatosis to NASH in humans^30^. Reduced CE might induce hepatocyte senescence as deduced from cell culture experiments^2^. It is thus plausible that LKO hepatocytes underwent a cell cycle arrest (Fig. 7, Table 1) due to diminished cholesterol reserves. Some LKOs also showed hepatic nuclear vacuolation, a proposed marker of hepatocyte senescence^31^. Supplementation with dietary cholesterol alleviated the oval cell response and fibrosis (Supplementary Fig. 7c, Supplementary Table 1) while lanosterol and DHL remained moderately elevated, suggesting that reduced hepatic cholesterol reserves drive the oval cell response of LKOs. Inflammation and fibrosis closely associate with the oval cell reaction^32^,^33^. Our data support the mechanism of activated oval cells entering into extensive cross-talk with injured hepatocytes, Kupffer cells and hepatic stellate cells (HSC)^34^, which play a major role in the derangement of lipid homeostasis, insulin resistance, and fibrogenesis in NAFLD^33^. Hepatocytes and Kupffer cells secrete TGF-β1, a primary pro-fibrogenic cytokine. Kupffer cells are also the major source of TNF-α, while biliary cells produce PDGFs. These HSC-activating factors were up-regulated in LKOs (Table 1, Supplementary Table 2, 3) and hepatic fibrosis/HSC activation was among the top enriched pathways in both sexes. The up-regulated HSC-activating cytokines, activated plasmin system and cytokines from infiltrating immune cells, aid in amplifying the oval cell response. Hepatocytes arrested in the cell cycle also secrete the senescence-associated secretory phenotype (SASP) proteins (Fig. 7) composed of interleukins, chemokines, growth factors, proteases and their regulators as well as extra-cellular matrix proteins^35^ (up-regulated in LKOs, Table 1).

Sex differences are gaining deserved recognition in hepatology^36^, albeit underlying mechanisms remain controversial. As deduced from the microarray data, a major hallmark of sexual dimorphism in *Cyp51* LKOs was a stronger activation of the inflammatory/immune response in males, and a female-specific down-regulation of *Ppara* signaling and of anti-inflammatory *Adipor2*^37^. Female-biased amino acid metabolism might represent a direct response to hepatomegaly^38^. PPAR*α* signaling is a determinant of estrogen-mediated hepatic lipid and glucose homeostasis^39^,^40^. Taken together, the down-regulated estrogen receptor *Esr1* (Supplementary Table 3) and *Ppara* might be crucial for the sexually dimorphic response of *Cyp51* LKOs when dietary cholesterol is limited (Fig. 7). The down-regulation of *Adipor2* emphasizes the sex-biased cross-talk between the liver and adipose tissue and is in mice linked to accumulation of reactive oxygen species^37^.

Decreased supply of dietary fatty acids that serve as endogenous PPAR-*a* ligands^41^ lead to decreased fatty acid β-oxidation. Reduced weight gain, less adiposity and lower hepatic phytosterols indicate fat malabsorption^42^ in the LKOs (Fig. 1d, e, Fig. 3c). Moreover, reduced hepatic BA synthesis (down-regulated *Cyp7b1, Cyp8b1, Hsd17b4, Hsd3b7*) and altered composition of gallbladder BAs (reduced cholic acid) also indicate a diminished capacity of fat emulsification, especially in females (Fig. 4a, b; Table 1)^42^. This points to the essential role of hepatic cholesterol synthesis in adequate intestinal fat absorption. When cholesterol resources are limited, the intact hepatic synthesis is crucial for the females due to developmental^43^ and reproductive^40^ energy demands. In males the extra-hepatic organs might to a greater extent contribute to cholesterol synthesis, as indicated by elevated HDL cholesterol in male LKOs (Fig. 4c). The increased corticosterone further implies an altered energy homeostasis^44^, another male-specific adaptation to the lack of *Cyp51*.

An incremental change in dietary fats (HFnC diet) in the absence of dietary cholesterol restored abdominal adipose depots and improved liver pathology mainly in females. Dietary fats provoked steatosis in both sexes irrespective of the dietary cholesterol or the cholesterol synthesis capacity (Fig. 5b, Supplementary Table 1). Steatosis did not worsen liver injury in male LKOs and had beneficial effects in the female LKOs. This is in line with the recent observation that people with hepatic triglyceride accumulation (“good fat storers”) are protected from NASH^45^. Steatosis and NASH may represent two separate diseases, with different molecular causes. Our data indicate that the diminished hepatic cholesterol synthesis through sterol imbalance and ISR led to NASH-like features, while dietary fats (in the absence of dietary cholesterol and hepatic cholesterol synthesis) led to steatosis. Only when cholesterol was added to the diet, the NASH-like features of LKOs were ameliorated, again in accordance with the role of sterol balance in this pathology.

The compensatory changes in cholesterol homeostasis on the HFnC diet were unable to fully restore the hepatic cholesterol ester reserves (Fig. 6). In the absence of cholesterol and fats (LFnC diet), females better adapted their amino and fatty acid metabolism, thus conserving essential nutrients, which proved to be beneficial also in the high-fat environment without cholesterol (HFnC). It is plausible that here the females could have efficiently stimulated the extra-hepatic cholesterol homeostasis^46^ and they also possess a larger BA pool^47^, which might have improved the dietary fat uptake. As opposed to the LFnC diet, males relied more on the intact cholesterol synthesis to secure precursors for BA synthesis.

In conclusion, our data indicate that abrogated hepatic cholesterol synthesis could contribute to progressive fibrosis in patients with inborn errors of cholesterol synthesis and that these effects might extend to the whole organism through impaired intestinal fat absorption. Our *Cyp51* LKO model shows that steatosis appears with dietary fats in the absence of dietary cholesterol and its synthesis, while a block in the cholesterol pathway leads to NASH-like features. Thus, it might be useful to re-consider the application of statins in certain liver disease patients.

## Methods

### Ethics Statement

All experiments were performed in accordance with the relevant guidelines and regulations. The experiments were approved by the Veterinary Administration of the Republic of Slovenia (license numbers 34401-31/2011/4 and 34401-52/2012/3) and were conducted in agreement with the European Convention for the Protection of Vertebrate Animals used for Experimental and Other Scientific Purposes (ETS 123), as well as in agreement with the National Institute of Health guidelines for work with laboratory animals.

### Animals and Diets

Transgenic mice carrying floxed exons 3 and 4 of the *Cyp51* allele^16^ were crossed with *Alb-Cre* mice to produce hepatocyte-specific *Cyp51* KO mice (*Cyp51^flox^*^/*flox*^; *Alb-Cre* or LKO) with *Cyp51^flox^*^/*flox*^ (LWT) mice serving as controls. At 3 weeks of age the mice were randomly assigned to either a standard rodent diet (low-fat no-cholesterol, LFnC) or a high-fat diet without (HFnC) or with cholesterol (HFC) for 16 weeks. Blood, bile and organs were collected.

### Histology

Hematoxylin and eosin (H&E), Sirius red and immunohistochemistry of pan-cytokeratins were performed on formalin-fixed paraffin-embedded liver sections. Sudan III and CYP51 immunolabeling was done on liver cryosections.

### Biochemistry

Lipids, ALT, AST, corticosterone and TNF-α were measured in plasma by commercial kits. Liver mRNA was applied for gene expression analyses by qPCR and Affymetrix microarrays. Total hepatic proteins were used for western blotting. Liver sterols and gallbladder BAs were analyzed by gas chromatography/mass spectrometry.

### Statistics

Linear regression modeling and empirical Bayes smoothing were applied with the genotype, sex and diet as predictor variables.

Detailed protocols are provided in the Supplementary Information.

## Author Contributions

Study design: D.R., S.H.; Study supervision: D.R.; Experiment conduct: G.L., M.P., R.K., M.L., F.M.G.M.; Data evaluation and interpretation: G.L., M.P., J.J., D.R.; Statistical analysis: P.J.; Material support: S.H., R.G., I.B.; Writing of the manuscript: G.L., D.R. All the authors contributed to the final version of the manuscript.

## Supplementary Material

Supplementary Information Supplementary information

Supplementary Information Supplementary tables

## Figures and Tables

**Table 1:** The most relevant enriched KEGG pathways and corresponding differentially expressed genes in LKO mice on standard laboratory diet (LFnC)

Females			
Enriched KEGG pathway	P value	Up-regulated DE genes	Down-regulated DE genes
Up-regulated			
Cell cycle	0.009	*Anapc2, Anapc4, Atr, Cdc14a, Cdkn1a, Dbf4, Gsk3b, Mad2l2, Sfn, Smad3, Tgfb1, Tgfb2, Tgfb3, Ywhah*	*Ccnd3, Cdc26, Rbl2*
Apoptosis	<0.001	*Apaf1, Bax, Bcl2, Bid, Birc3, Capn1, Capn2, Casp12, Csf2rb2, Endod1, Ikbkg, Irak3, Map3k14, Ngf, Pik3r5, Prkx, Rela, Tnf, Tnfrsf10b*	*Aifm1, Akt2, Chuk, Dffa, Il1rap*
TGF-β signaling pathway	0.007	*Bmp4, Gdf6, Mapk3, Ppp2r1a, Rock2, Smad1, Smad3, Tgfb1, Tgfb2, Tgfb3, Tgfbr1, Tgfbr2, Thbs1, Tnf*	*Acvr2b, Amh, Rbl2*
Senescence- associated secretory phenotype	Compiled from^35^	*Ccl3, Col1a1, Col1a2, Col3a1, Col6a3, Csf2ra, Csf2rb2, Csf3r, Cxcl5, Cxcr4, Icam1, Igfbp7, Il6st, Il7r, Kitl, Lama5, Lamc2, Mmp12, Mmp13, Mmp14, Ngf, Plat Plau, Plaur, Serpine1, Timp1, Timp2*	*Igfbp3, Il6ra, Il13, Lama1*
Integrated stress response	Compiled from^22^	*Eif2ak3, Eif4e3, Atf3, Eif4g3, Hmox1, Slc3a2, Slc6a9, Sqstm1*	*Mthfd1, Nfe2l1*
Down-regulated			
Primary bile acid biosynthesis	0.01		*Amacr, Hsd17b4, Hsd3b7*
Peroxisome	<0.001	*Acox3, Xdh*	*Acaa1a, Acaa1b, Acot8, Acox1, Acsl1, Agxt, Amacr, Decr2, Dhrs4, Ech1, Eci2, Gstk1, Hacl1, Hao1, Hao2, Hmgcl, Hsd17b4, Mlycd, Paox, Pecr, Pex1, Pex6, Pex10, Pex11a, Pex11c, Pex13, Pex14, Pex16, Pex3, Pex7, Pxmp2, Pxmp4, Slc27a2*
Fatty acid metabolism	<0.001	*Acox3*	*Acaa1a, Acaa1b, Acaa2, Acadl, Acads, Acadsb, Acadvl, Acat1, Acox1, Acsl1, Adh5, Aldh9a1, Cpt2, Echs1, Eci1, Eci2, Gcdh, Hadh, Hadha*
Adipocytokine signaling pathway	0.014	*Ikbkg, Jak2, Nfkbib, Nfkbie, Ptpn11, Rela, Slc2a1, Tnf*	*Acsl1, Adipor2, Akt2, Chuk, G6pc, Lepr, Ppara, Prkag2, Rxra, Stk11*
Alanine, aspartate and glutamate metabolism	0.002	*Gfpt2, Gls*	*Abat, Acy3, Agxt, Aldh4a1, Asl, Aspa, Got1*
Arginine and proline metabolism	0.016	*Abp1, Aldh18a1, Ckb, Gls, P4ha2, Sat1*	*Acy1, Agmat, Aldh4a1, Aldh9a1, Arg1, Asl, Gamt, Got1, Prodh2, Pycrl*
Valine, leucine and isoleucine degradation	<0.001	*Oxct1*	*Abat, Acaa1a, Acaa1b, Acaa2, Acad8, Acads, Acadsb, Acat1, Aldh6a1, Aldh9a1, Auh, Bckdha, Dbt, Echs1, Hadh, Hadha, Hibch, Hmgcl, Hmgcs2, Ivd, Mccc1, Mccc2, Pcca*

Males			
Enriched KEGG pathway	P value	Up-regulated DE genes	Down-regulated DE genes
Up-regulated			
Cell cycle	0.017	*Atr, Ccnd2, Cdc14a, Cdc23, Cdkn1b, Cdkn2c, E2f3, Gsk3b, Mad2l1, Orc4, Rb1, Rbl1, Smad2, Tgfb2, Tgfb3, Ywhah*	
Apoptosis	0.001	*Akt3, Apaf1, Bcl2, Birc2, Birc3, Casp12, Csf2rb, Csf2rb2, Endod1, Il1b Irak3, Irak4, Map3k14, Nfkb1, Ngf, Pik3cg, Pik3r3, Ppp3cc, Prkar1b, Prkar2b, Prkx, Tnf, Tnfrsf10b*	*1500003O03Rik, Dffa, Endog*
Senescence- associated secretory phenotype	Compiled from^35^	*Ccl3, Col1a1, Col14a1, Col15a1, Col1a2, Col3a1, Col4a2, Col6a1, Col6a3, Col8a1, Csf2rb, Csf2rb2, Csf3r, Cxcl5, Cxcr4, Hgf, Icam1, Igfbp7, Il1b, Il7r, Kitl, Lama2, Lama4, Lama5, Lamb1, Lamc2, Mmp12, Mmp13, Ngf, Plat, Timp1, Timp2, Vegfc*	*Ccl26, Col10a1, Col17a1, Col18a1, Cxcl13, Igfbp4, Il15ra, Il6ra, Mmp10*
Integrated stress response	Compiled from^22^	*Eif2ak3, Eif2ak4, Eif2s1, Eif4e3, Nfe2*	*Eif3c, Ern2*
Terpenoid backbone synthesis	0.001	*Acat2, Fdps, Hmgcr, Hmgcs1, Mvd, Mvk, Pmvk*	
Down-regulated			
Primary bile acid biosynthesis	0.048		*Amacr*

Differentially expressed genes related to senescence-associated secretory phenotype and integrated stress response were compiled from Refs. 35 and 22, respectively. Depending on the direction of expression of the majority of DE genes, the parametric gene set enrichment analysis defines enriched KEGG pathways as up- or down-regulated. DE, differential expression; KEGG, Kyoto Encyclopedia of Genes and Genomes.
