# Supplementary material for: Lessons from Hepatocyte-Specific Cyp51 Knockout Mice: Impaired Cholesterol Synthesis Leads to Oval Cell-Driven Liver Injury
Source: Sci Rep. 2015 Mar 5;5:8777. doi: 10.1038/srep08777 (PMC4350092; doi:10.1038/srep08777)
Supplement: Supplementary Information [file srep08777-s1.pdf]

Supplementary Information

Lessons from Hepatocyte-Specific *Cyp51* Knockout Mice: Impaired Cholesterol Synthesis Leads to Oval Cell-Driven Liver Injury

Gregor Lorbek, Martina Perše, Jera Jeruc, Peter Juvan, Francisco M. Gutierrez-Mariscal, Monika Lewinska, Rolf Gebhardt, Rok Keber, Simon Horvat, Ingemar Björkhem, and Damjana Rozman

Abbreviations

LKO, *Cyp51*$^{flox/flox}$;*Alb-Cre* or liver *Cyp51* knockout mice; LWT, *Cyp51*$^{flox/flox}$ or control mice; (q)PCR, (quantitative) polymerase chain reaction; LFnC, low-fat no-cholesterol diet; HFnC, high-fat diet without cholesterol; HFC, high-fat diet with cholesterol; ALT, alanine aminotransferase; AST, aspartate aminotransferase; H&E, hematoxylin and eosin; RT, room temperature; GC/MS, gas chromatography/mass spectrometry; BA, bile acid; DE, differential expression; FDR, false discovery rate; KEGG, Kyoto Encyclopedia of Genes and Genomes; pGSEA, Parametric Gene Set Enrichment Analysis; GEO, Gene Expression Omnibus

Supplementary materials and methods

Animals

The generation of transgenic mice carrying floxed exons 3 and 4 of the *Cyp51* allele was reported previously[1]. To establish hepatocyte-specific deletion, $Cyp51^{flox/flox}$ mice on a mixed genetic background (129/Pas (10%) × C57BL/6J (90%)) were crossed with transgenic mice expressing Cre recombinase under the rat albumin promoter (*Alb-Cre*). $Cyp51^{flox/flox}$;*Alb-Cre* mice in this study were considered as liver knockout (LKO) and $Cyp51^{flox/flox}$ as control (LWT) mice. Genotypes were confirmed by polymerase chain reaction (PCR) of tail gDNA as described[1]. During the experiment the mice were subjected to routine microbiological monitoring where some (both genotypes) tested positive for murine norovirus and hepatitis virus antigens. All procedures were conducted in accordance with the National Institute of Health guidelines for work with laboratory animals and the European Convention for the Protection of Vertebrate Animals used for Experimental and Other Scientific Purposes (ETS 123), and were approved by the Veterinary Administration of the Republic of Slovenia (license numbers 34401-31/2011/4 and 34401-52/2012/3).

Diet Regime

LKO and LWT mice were bred at the Medical Experimental Centre, Faculty of Medicine, University of Ljubljana, Slovenia and maintained in a temperature and humidity controlled environment under a 12h light/dark cycle (lights were on from 7.00 am to 7.00 pm). Animals had *ad libitum* access to food and acidified tap water (pH = 3). After the weaning period, 3-week LKO and LWT mice of both sexes were randomly assigned to one of the three diets: standard laboratory chow (1324, Altromin, Lage, Germany) regarded as a low-fat no-cholesterol diet (LFnC); high-fat diet without cholesterol (HFnC) (D12108C, Research Diets,

Inc., New Brunswick, NJ, USA) and high-fat diet with 1.25% (w/w) of cholesterol (HFC) (D12106C, Research Diets, Inc., New Brunswick, NJ, USA). A total of 136 mice (9 to 13 animals per group) were fed for 16 weeks and were weighed weekly. As a final body weight a four-week average weight prior to euthanasia was used in order to reduce daily and weekly variations[2]. Weight at euthanasia was used to calculate organ to body weight ratios. The summary of the diets is provided in the Table SM1.

Table SM1: Summary of used diets

Diet	**Low-fat no cholesterol diet (LFnC)**	**High-fat diet without cholesterol (HFnC)**	**High-fat diet with 1.25% cholesterol (HFC)**
	Maintenance diet – rats and mice	Clinton/Cybulsky High Fat Rodent Diet	Clinton/Cybulsky High Fat Rodent Diet
Manufacturer	Altromin	Research Diets Inc.	Research Diets Inc.
Item number	1320	D12106C	D12108C
Energy (kcal/kg)	2844	4056	4056
Proteins	24 kcal %	20 kcal %	20 kcal %
Carbohydrates	65 kcal %	40 kcal %	40 kcal %
Fat	11 kcal %	40 kcal %	40 kcal %

Euthanasia and Sample Collection

Mice were euthanized after a 5 h fast with cervical dislocation at the same time of the light period (11.30 am – 3.00 pm) in order to avoid diurnal variability as much as possible. Blood was collected into heparin coated Vacuette MiniCollect® 1 ml Plasma Tubes (Greiner Bio-one, Frickenhausen, Germany) by a left ventricle heart puncture. Heart, kidneys, spleen, liver and abdominal fat depots (epididymal, retroperitoneal and mesenteric) were removed and weighed. Bile was removed from the gallbladder with a syringe and snap-frozen in liquid nitrogen together with kidneys and part of livers that were cut to thin slices. Plasma was

prepared by blood centrifugation at 4° C, 3000 g for 15 min. All the material was stored at -80° C for subsequent analysis.

Plasma Biochemistry

Total cholesterol (7D62, Abbott Laboratories, Abbott Park, IL, USA), HDL cholesterol (3K33, Abbott Laboratories, Abbott Park, IL, USA), LDL cholesterol (61534, BioMérieux, Marcy l'Etoile, France), triglycerides (7D74, Abbott Laboratories, Abbott Park, IL, USA), free fatty acids (436-91995, Wako Pure Chemical Industries, Osaka, Japan), alanine aminotransferase (ALT) (8L92-40, Abbott Laboratories, Abbott Park, IL, USA) and aspartate aminotransferase (AST) (8L91-41, Abbott Laboratories, Abbott Park, IL, USA) were analyzed by Veterinarska ambulanta BTC (Ljubljana, Slovenia) with Architect ci8200 analyzer (Abbott Diagnostics, Abbott Park, IL, USA). 7-8 randomly chosen samples per group were analyzed. Concentrations of lipid parameters are given in mmol/l and the activity of aminotransferases in µkat/l.

Corticosterone was measured in 6 samples from each group with the Corticosterone (Rat/Mouse) ELISA kit (EIA-5186, DRG Instruments, Marburg, Germany). 4 samples per sex/genotype in the LFnC diet group and 3 samples in the HFnC and HFC diet groups were used for TNF-α measurements with the TNF-α (Mouse) ELISA kit (EIA-5692, DRG Instruments, Marburg, Germany) according to the manufacturer's instructions. All measurements were done in duplicates. Average absorbance values were used to calculate the concentrations using the Four Parameter Logistic curve fitting with the GraphPad Prism 5™ software (GraphPad Software, Inc., La Jolla, CA, USA). Values for corticosterone are given in ng/ml and for TNF-α in pg/ml.

Histology and Immunohistochemistry

Left lateral liver lobes were taken for histological analyses. Half of the lobes were fixed in 4% phosphate-buffered formalin, embedded in paraffin and stained with hematoxylin and eosin (H&E) (standard histological evaluation), 0.1% Sirius red (365548, Sigma-Aldrich, St. Louis, MO, USA) in saturated picric acid (evaluation of fibrosis) and immunolabeled for pan-cytokeratins (wide-spectrum cytokeratins as markers for oval cells and cholangiocytes). The other half of the liver lobes were embedded in Jung tissue-freezing medium (Leica Microsystems, Nussloch, Germany), slowly frozen in liquid nitrogen for cryosection preparation, cut to 3-4 μm slides and stained with Sudan III (evaluation of steatosis) or immunolabeled for CYP51. All slides were evaluated by a pathologist (J. J.) blinded to the study.

HE stained liver sections were scored for different parameters such as size, shape and polymorphism of hepatocytes and their nuclei; presence of nucleoli/nuclear vacuolization; presence and location of inflammatory cells; cholestasis; steatosis; apoptosis; mitosis and potential abnormalities of bile ducts.

The extent of cholestasis (in the cytoplasm of hepatocytes or in the lumen of bile ducts) was scored as none (0) when no cholestasis was observed; mild (+) when rarely observed; moderate (++) when present in more than 3 areas of the section and severe (+++) when widespread. The percentage of hepatocytes containing lipid droplets was used as a scoring system for steatosis (micro or macrovesicular): no steatosis (less than 5%); mild (+) (5-25%); moderate (++) (25-50%); severe (+++) (50-75%) and very severe (++++) (more than 75%). Apoptoses and mitoses were counted on the whole liver section. Liver samples were taken in the same way for every mouse (vertical cut in the middle of the left lobe) to exclude variability due to the technical preparation of the sample. Apoptotic cells were recognized by characteristic morphological features, such as chromatin condensation, cell shrinkage and

nuclear fragmentation[3]. Mitotic figures were identified according to the criteria proposed by van Diest *et al.*[4]. Only cells with clear morphological features of metaphase, anaphase, and telophase were counted, avoiding apoptotic and hyperchromatic nuclei. The extent/intensity of bile ducts proliferation (oval cell response/ductular reaction) was scored as follows: normal (0; no proliferation observed); mild (+) when more than two bile ducts were seen in the portal field or were extending to the middle zone with or without bridging; moderate (++) when more than 5 bile ducts were observed periportaly extending to the middle zone with or without bridging; severe (+++) when bile ducts were extending from one portal field to another – bridging. Stages of fibrosis were assessed on Sirius red stained slides as follows; stage 0 (no fibrosis observed); stage 1 (+) (periportal fibrosis); stage 2 (++) (extension of periportal fibrosis to the middle zone without bridging); stage 3 (+++) (bridging fibrosis) and stage 4 (++++) (cirrhosis).

To confirm the bile duct proliferation, immunohistochemical staining for pan-cytokeratins was performed. Deparaffinized and rehydrated slides were subjected to heat induced epitope retrieval in 0.01 M citrate buffer (pH = 6) in a microwave oven for 20 min. After cooling down to room temperature, blocking for endogenous peroxidases was performed with 3% H_2O_2 for 10 min. Prior to overnight incubation at 4° C with the 1:1000 diluted anti-cytokeratin antibody (Z0622, Agilent Technologies DAKO, Glostrup, Denmark), slides were blocked with 5% goat serum (G9023, Sigma-Aldrich, St. Louis, MO, USA) for 30 min at room temperature (RT). Following incubation with the biotinylated goat anti-rabbit secondary antibody (AP132B, Millipore, Billerica, MA, USA) (1:1000 dilution) for 1h at RT, incubation with Extravidin®-Peroxidase (E2886, Sigma-Aldrich, St. Louis, MO, USA) (1:1000 dilution) was performed for 30 min at RT. 0.5 mg/ml of Diaminobenzidine (D5637, Sigma-Aldrich, St. Louis, MO, USA) was used as a chromogen. The slides were then

counterstained with Mayer's Hematoxylin (MHS16, Sigma-Aldrich, St. Louis, MO, USA) for 5 min, dehydrated in grading ethanols and coversliped.

In order to evaluate the extent and success of CYP51 deletion in hepatocytes, immunolabeling for CYP51 was performed. Cryosection slides were air-dried for 10 min at RT and fixed in ice-cold acetone for 10 min. Endogenous peroxidases were blocked with 0.3% H_2O_2 for 10 min and incubation with 5% bovine serum albumin (A3294, Sigma-Aldrich, St. Louis, MO, USA) was performed for another 30 min at RT. Blocking for endogenous biotin was done with 0.001% avidin and 0.001% biotin (both Sigma-Aldrich, St. Louis, MO, USA) for 15 min each at RT. Incubation with the primary anti-CYP51 antibody (see Table SM2) (1:300 dilution) was done on 4° C overnight. Incubation with the secondary antibody (B8895, Sigma-Aldrich, St. Louis, MO, USA) (dilution 1:600) and all subsequent steps were done as described above.

RNA and DNA isolation

RNA isolation and cDNA synthesis were done as previously described[5]. Genomic DNA for determining *Cyp51* excision efficiency was isolated from 5 liver and from 3 kidney samples per group in the LFnC diet only. The isolation of gDNA was performed with a standard phenol:chloroform:isoamyl alcohol (25:24:1) (77617, Sigma-Aldrich, St. Louis, MO, USA) extraction procedure using Proteinase K (V302B, Promega, Madison, WI, USA) to lyse the tissue. gDNA concentration and purity were assayed with the use of NanoDrop 1000 Spectrophotometer (Thermo Fisher Scientific, Waltham, MA, USA). Prior to the analysis, the gDNA was diluted to a final concentration of 16 ng/µl.

Real-time Quantitative Polymerase Chain Reaction (qPCR)

Real-time qPCR analysis of mRNA was done according to[5]. 5 samples per group were analyzed. To determine the excision efficiency of the *Cyp51* gene by Cre recombinase, we designed primers that detect the *Cyp51^{flox}* allele, but fail to amplify the *Cyp51*-null allele (*Cyp51^{flox}*-Fw: AATGCTGAGAGCAACGGACT; *Cyp51^{flox}*-Rv: TCCCACCTCCTGTCTTCATC). The PCR reaction consisted of 0.75 μL of template gDNA, 0.6 μL of 2.5 μM primer mix, 2.5 μL of SYBR Green I Master (Roche, Basel, Switzerland) and 1.15 μL of RNAse-free water in a final volume of 5 μL. Three technical replicates were performed for each sample. The following thermal cycling conditions were used: 10 min at 95° C followed by 45 cycles of 10 s at 95° C, 20 s at 58° C and 20 s at 72° C. The amounts of the *Cyp51^{flox}* allele were normalized to the expression of β-actin (*Actb*-Fw: AGAGGGAAATCGTGCGTGC; *Actb*-Rv: CAATAGTGATGACCTGGCCGT).

Total Protein Isolation and Western Blot Analysis

Liver samples were homogenized in a lysis buffer containing 20 mM Tris/HCl (pH 7.5), 0.15 M NaCl, 1% NP-40, 10% glycerol, 5 mM EDTA and 1 mM PMSF supplemented with the cOmplete protease inhibitors cocktail (Roche, Basel, Switzerland). The extracts were incubated at 4° C for 2 hours followed by centrifugation at 12.000 g, 4° C for 15 min. Supernatants containing the protein extracts were quantified using the Pierce™ BCA Protein Assay Kit (Thermo Fisher Scientific, Waltham, MA, USA). 40 μg of total liver proteins were separated on 10% SDS-PAGE gels and subsequently electro-transferred onto PVDF membranes (Immobilon-P Membranes, Millipore, Billerica, MA, USA). All used primary antibodies that are specified in the Table SM2 were rabbit polyclonal antibodies and were subsequently incubated with the respective goat anti-rabbit secondary antibody (1:10000) (A0545, Sigma-Aldrich, St. Louis, MO, USA). The development process was carried out with

Luminol Enhancer Solution (Thermo Fisher Scientific, Waltham, MA, USA). The relative amount of each protein was quantified by densitometry using the Quantity-One™ software (Bio-Rad Laboratories, Hercules, CA, USA). Values were normalized to the intensity of GAPDH and expressed in arbitrary units (AU).

Table SM2: Primary antibodies used for immunohistochemistry and western blots

Protein	Company	Product number	Dilution
HMGCR	Millipore, Billerica, MA, USA	ABS229	1:1000
CYP51	Custom made	against the peptide QRLKDSWAERLDFN PDRY	1:500
DHCR14/*Tm7sf2*	Proteintech Group, Inc., Chicago, IL, USA	12033-1-AP	1:500
SREBP2	Proteintech Group, Inc., Chicago, IL, USA	14508-1-AP	1:500
GAPDH	Sigma-Aldrich, St. Louis, MO, USA	G9545	1:500

Total Sterol and Bile Acid Extraction and GC/MS Analysis

The extraction of total sterols from the liver and bile acids (BA) from the gallbladder bile as well as subsequent analyses with gas chromatography/mass spectrometry (GC/MS) were conducted as previously described[5]. From 8-10 samples in each group were analyzed. Values for cholesterol synthesis intermediates are given in ng/mg of wet liver tissue and BA are given in relative amounts (%).

Microarray Hybridization and Data Analysis

3 samples per group were randomly selected for analysis with Affymetrix Gene Chip Mouse 1.0 ST Arrays (Affymetrix, Santa Clara, CA, USA). 150 ng of total RNA that met the required thresholds of purity (absorbance ratio A260/280 > 1.8) and quality (RIN > 8.5) was

used for hybridization to the microarrays according to the manufacturer's instructions. After overnight hybridization the arrays were stained and washed on GeneChip Fluidics Station 450 and scanned on Affymetrix GeneChip Scanner 3000 7G. Images were analyzed by Affymetrix Expression Console™ version 1.0 and all passed the software's quality control assessment. Further data analysis was performed with different R/Bioconductor software packages. Additional quality control and evaluation for potential outliers was utilized with the arrayQualityMetrics package, where also none of the samples was flagged as an outlier. Raw expression data was normalized using the RMA algorithm from the XPS package. Differential gene expression (DE) evaluation was done with the LIMMA package using the three predictor variables (diet, sex and genotype) and their interactions. False discovery rate (FDR) correction for multiple hypothesis testing with $\alpha = 0.05$ as a significance threshold was used. Euler diagram was created with the venneuler package. Gene annotation and data representation was done with the annaffy and affycoretools packages. Gene set enrichment analysis for Kyoto Encyclopedia of Genes and Genomes (KEGG) pathways was performed using the Parametric Gene Set Enrichment Analysis (pGSEA) package in combination with the LIMMA package by setting the statistical significance threshold to $p = 0.05$ and applying the FDR correction. For each contrast of interest representing differences between genotypes, sex and diet regimes, a list of DE genes and enriched KEGG pathways was generated. For the analysis with the Ingenuity Pathway Analysis™ platform, a cutoff of 1.5 fold change difference in gene expression was applied. For the generation of interaction network maps, the same lists of DE genes for males and females were mapped to the "experiment" and "database" libraries of the STRING online software[6]. Confidence score was set to medium (0.4). The interaction network maps were visualized using Cytoscape in an organic layout[7].

The raw and normalized gene expression data together with the experimental information was deposited to the Gene Expression Omnibus (GEO) database under the accession number GSE58271.

Statistical Analysis

Statistical analyses were performed using the linear regression modeling and empirical Bayes smoothing of the standard errors with the LIMMA package in the R environment. Three predictor variables corresponding to the genotype (LKO or LWT), sex (F or M) and diet (LFnC, HFnC or HFC) together with their interactions were used to estimate gross mouse characteristics, gene expression values, protein amounts, plasma parameters, liver sterol levels and relative amounts of bile acids. The gene expression and metabolites of cholesterol and bile acid synthesis revealed interesting sexual dimorphism on the control (wild type) LWT mice[5]. This set of LWT data was included into a broader statistical analysis for comparison to the LKO data. Several potential confounding variables were considered in the statistical model, such as the exact time of euthanasia, genotype of the mother and father, size of the litter from which the mouse originated, and mouse age at the start of the diet. The consensus correlation coefficient over all variables showed that their effects on the total variance were negligible and were therefore excluded from the statistical model. The Yeo-Jahnsen family of transformations[8] from the car package was applied to individual response variables in order to improve normality of their distribution; however, the non-transformed data were used for graphical and tabular representation in the manuscript.

Linear model fitting using the three predictor variables was thus applied to the transformed data and FDR correction was used for multiple hypothesis testing to discover statistically significant differences in the response variables at the threshold of $p < 0.05$ (marked as *) and $p < 0.1$ (marked as $^{\bullet}$).

Supplementary Figures

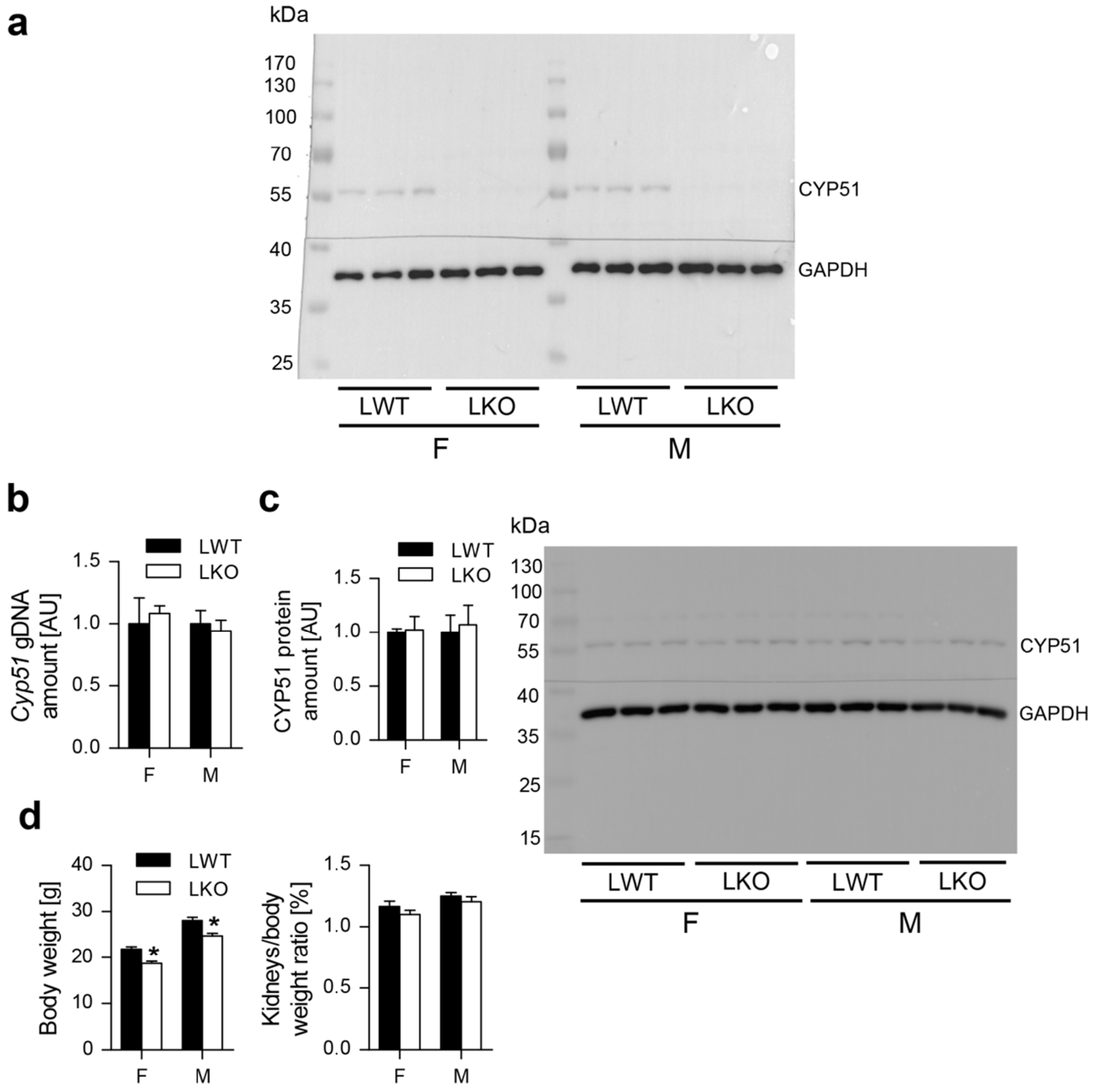

Supplementary Figure 1: *Cyp51* status in liver and kidneys together with body weight and kidneys weight ratio. (a) Uncropped Western blot of CYP51 in the liver. The membrane was cut in order to stain each section with the respective antibody. GAPDH was used as a loading control. **(b)** Relative *Cyp51* gDNA amount of the LWT and LKO mice (n = 3) in the kidneys as determined by qPCR. **(c)** Western blot analysis of CYP51 in the kidneys for females and males of both genotyes (n = 3) and a corresponding relative quantification graph. The membrane was cut in order to stain each section with the respective antibody. GAPDH

was used as a loading control. **(d)** Body weight and kidneys weight ratio of the mice of both genotypes and sexes (n = 9 - 13). Columns represent means and error bars represent SEMs. AU – arbitrary units. * $p < 0.05$.

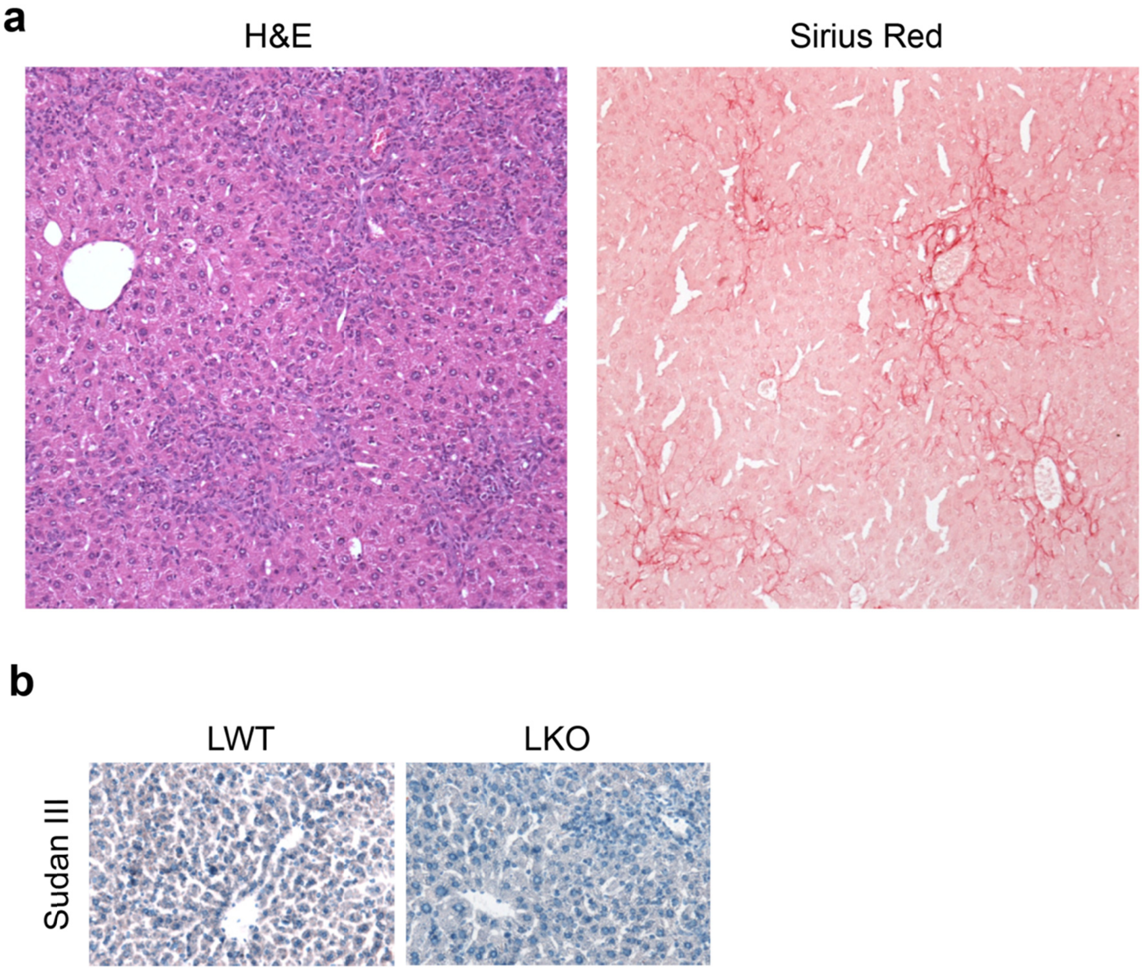

Supplementary Figure 2: Extent of ductular reaction, fibrosis and steatosis in LKO mice on low-fat no-cholesterol diet. (a) Representative low-magnification photomicrographs of the H&E and Sirius red staining in the LKO samples on the LFnC diet. Original magnification 100×. **(b)** Representative Sudan III staining for the LWT and LKO mice on the LFnC diet. Original magnification 200×.

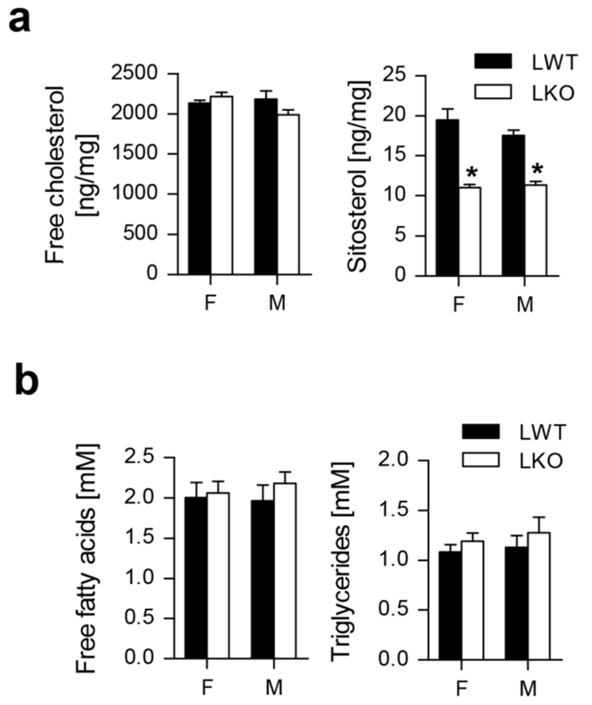

Supplementary Figure 3: Hepatic and blood LKO lipid homeostasis on the low-fat no-cholesterol diet. (a) Hepatic concentrations of free cholesterol and sitosterol of the LWTs and LKOs on the LFnC diet (n = 8-9). **(b)** Levels of triglycerides and free fatty acids on the standard LFnC diet in plasma (n = 8). Columns represent means and error bars represent SEMs. * $p < 0.05$.

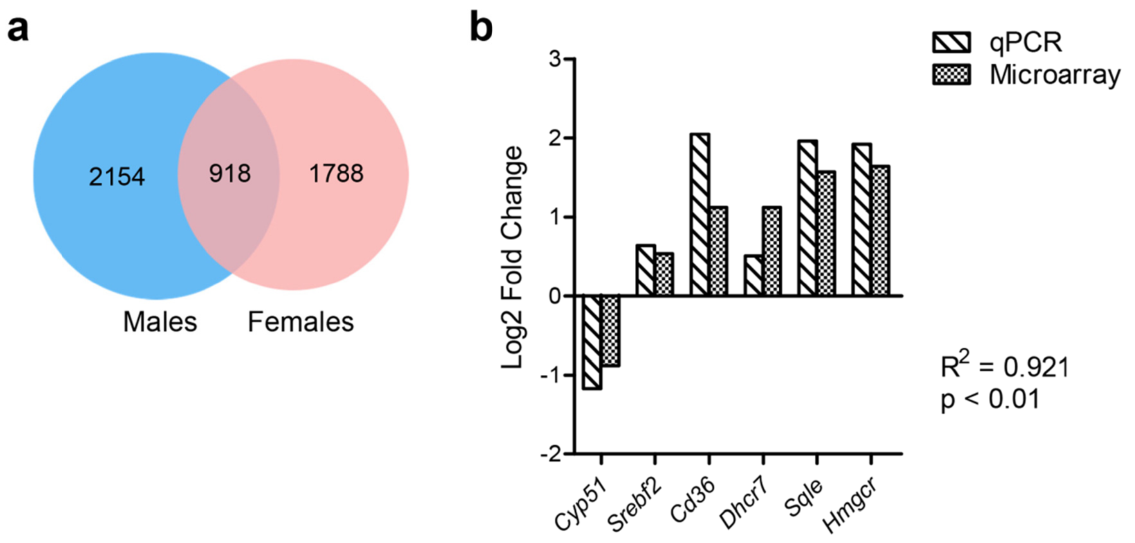

Supplementary Figure 4: Euler diagram of male *vs* female comparison of differentially expressed genes (a) and validation of microarray data by qPCR (b). Genes from the cholesterol synthesis and homeostasis of the male LKO mice on the LFnC diet were used (n = 5). Log2 fold change for *Cyp51* on microarrays was only marginally statistically significant and is not shown in Table 1 or in supplementary tables for males. Pearson's correlation coefficient between qPCR and microarray data is shown on the graph.

a

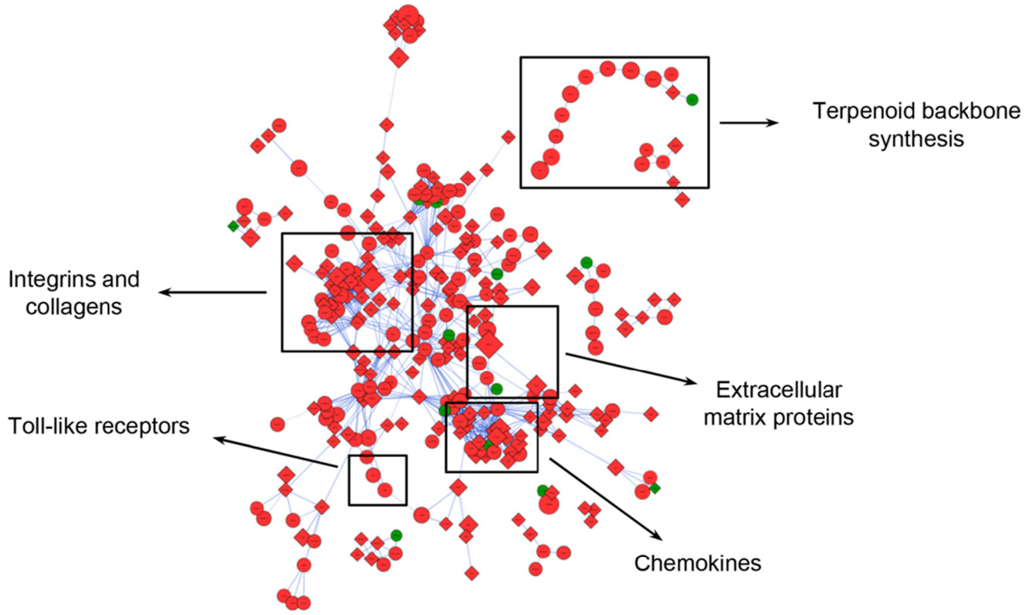

b

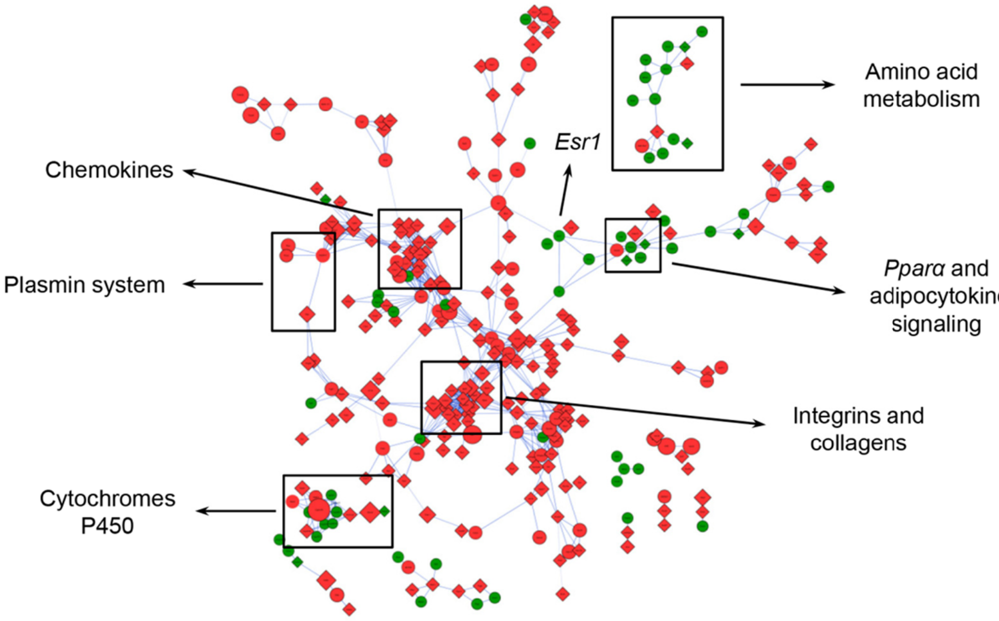

Supplementary Figure 5: Interaction network maps of differentially expressed genes for male (a) and female (b) LKO mice. Differentially expressed genes (1.5-fold change threshold) were mapped on the STRING database and interaction networks were constructed based on experimentally defined protein-protein interactions. Male interaction network is more uniform and mainly related to the up-regulated immune response. From the primary metabolic pathways only cholesterol synthesis is specifically up-regulated in the males. Female interaction network is on the other hand more diversified, with more down-regulated

clusters, such as amino acid metabolism or some cytochromes P450. Important gene clusters are highlighted. Up-regulated genes are labelled red and down-regulated genes green. Circles represent female- and male-specific gene expression, whereas diamond shapes represent common genes. Size of the node represents fold-change. Interactions of only two genes that are not connected to the main interaction network are not shown.

a

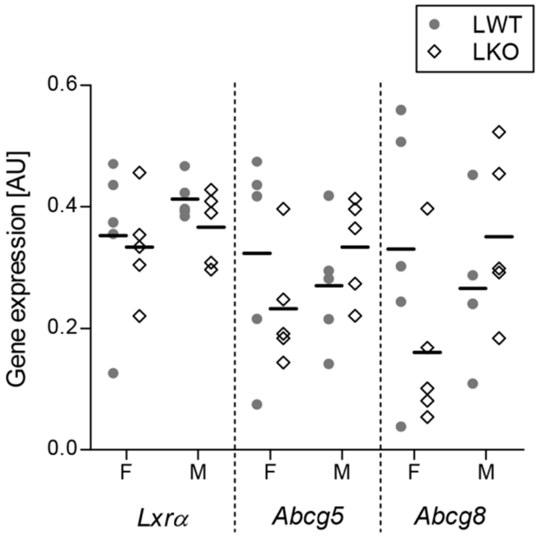

b

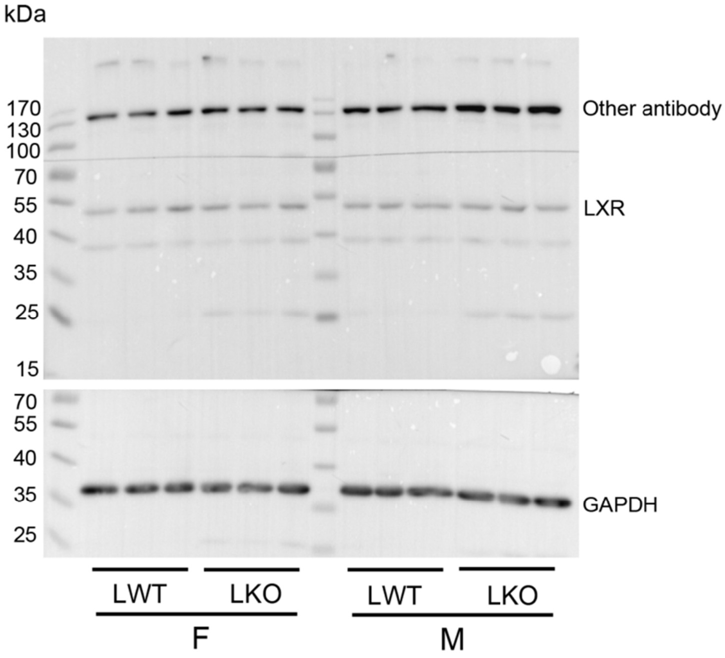

Supplementary Figure 6: Gene expression and protein analysis of LXR and its downstream target genes. (a) Gene expression of *Lxrα* and its two downstream target genes *Abcg5* and *Abcg8* on the low-fat no-cholesterol diet. **(b)** Western blot analysis of LXR. The membrane was cut in order to stain each section with the respective antibody. GAPDH was used as a loading control. AU – arbitrary units.

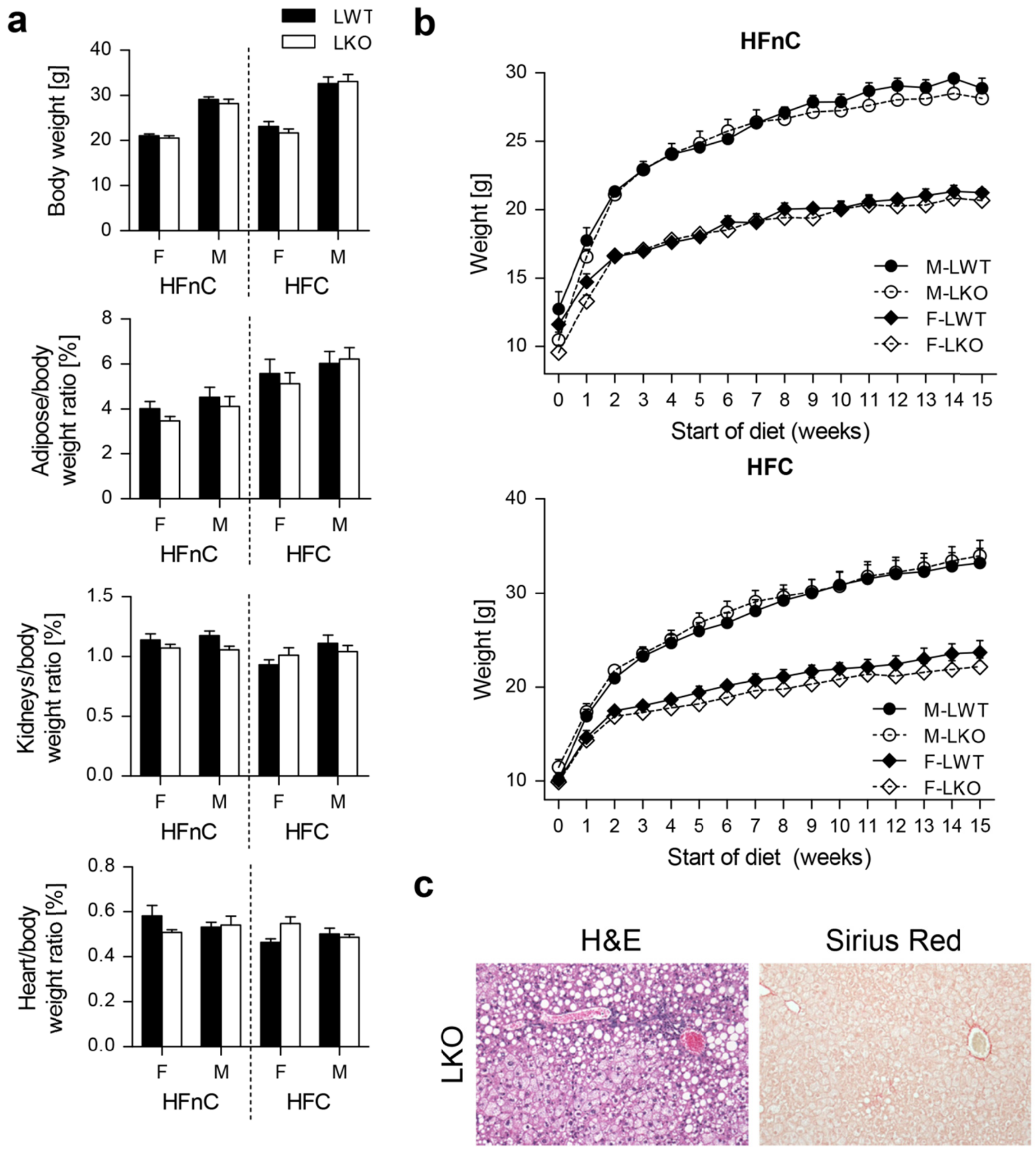

Supplementary Figure 7: Gross phenotype and liver histology of LKO mice on both high-fat diets. (a) Body weight and different organ to body weight ratios of the LWT and LKO mice on both high-fat diets (n = 10-13). **(b)** Growth curves of the mice on high-fat diets (n = 10-13). **(c)** Representative photomicrographs of the LKO mice on the HFC diet. Original magnification 100×. Columns represent means and error bars represent SEMs. HFnC, high-fat diet without cholesterol; HFC, high-fat diet with cholesterol.

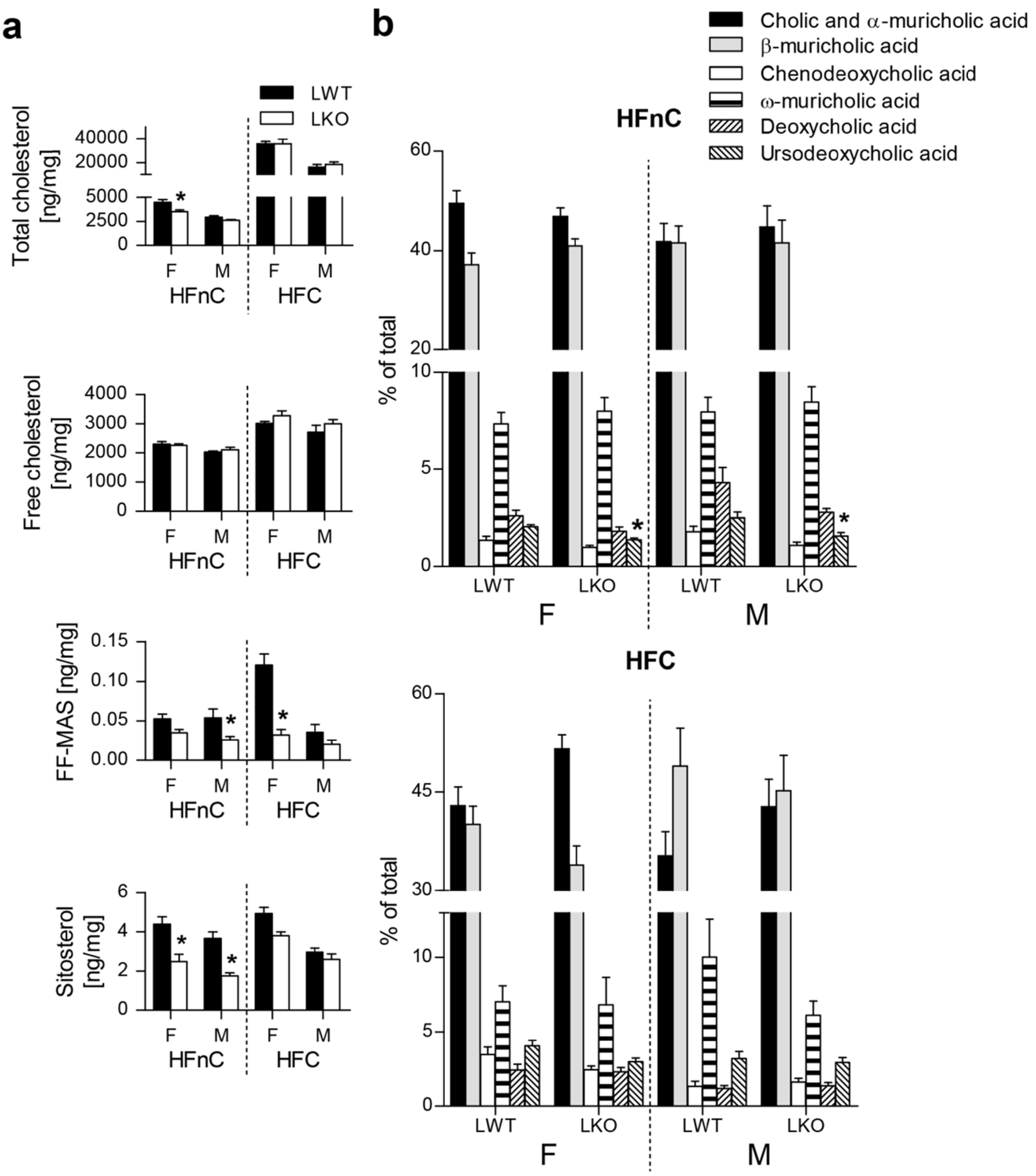

Supplementary Figure 8: Hepatic levels of remaining sterols on HFnC and HFC diets together with gallbladder bile acid composition. (a) Hepatic levels of total and free cholesterol and sitosterol in both sexes on the HFnC and HFC diets (n = 8-10). **(b)** Gallbladder bile acid composition on both high-fat diets (n = 7-9). Columns represent means and error bars represent SEMs. HFnC, high-fat diet without cholesterol; HFC, high-fat diet with cholesterol. * $p < 0.05$.

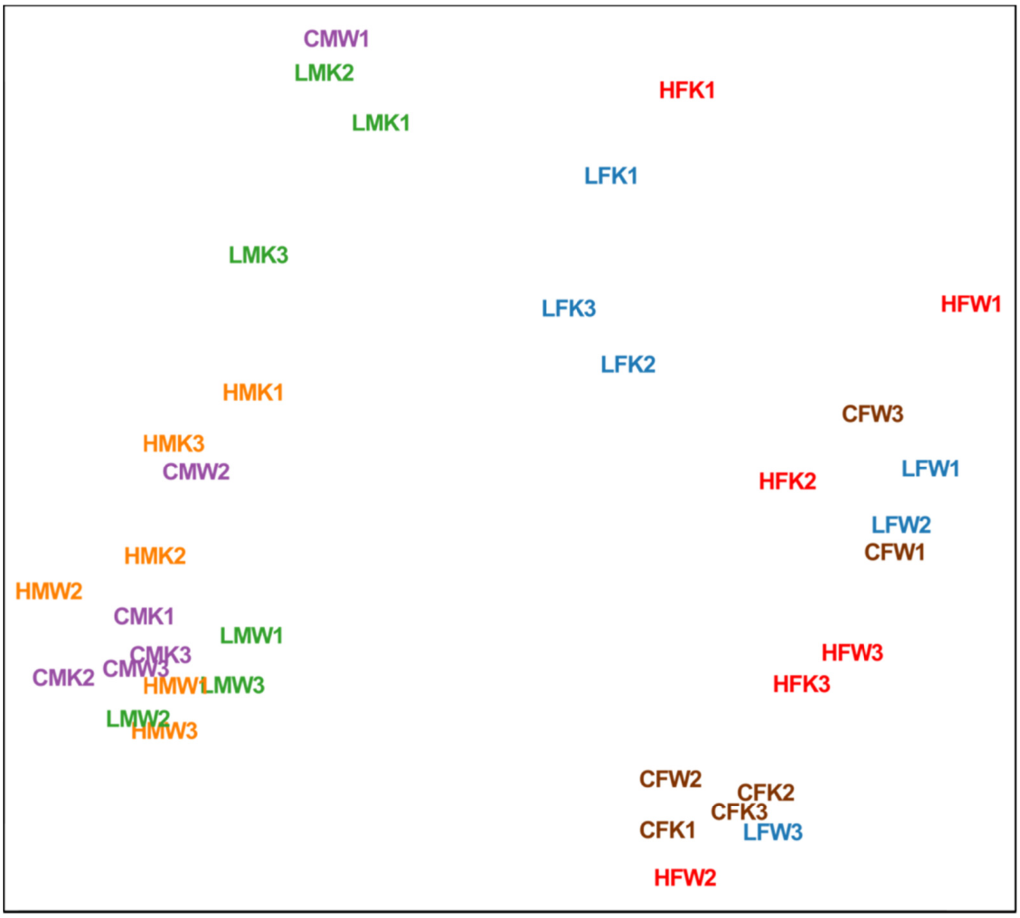

Supplementary Figure 9: Multi-dimensional scaling of the microarray samples according to the 100 most variable Affymetrix probes. Samples are colored by diet and sex only, meaning that both genotypes inside the same diet and sex have identical color. The three-letter coding indicates (1) the diet, (2) sex and (3) genotype, and the number indicates biological replica. (1) L – low-fat no-cholesterol diet, H – high-fat no-cholesterol diet, C – high-fat diet with cholesterol; (2) M – males, F – females; (3) W – liver control or LWT, K – liver knockout or LKO.

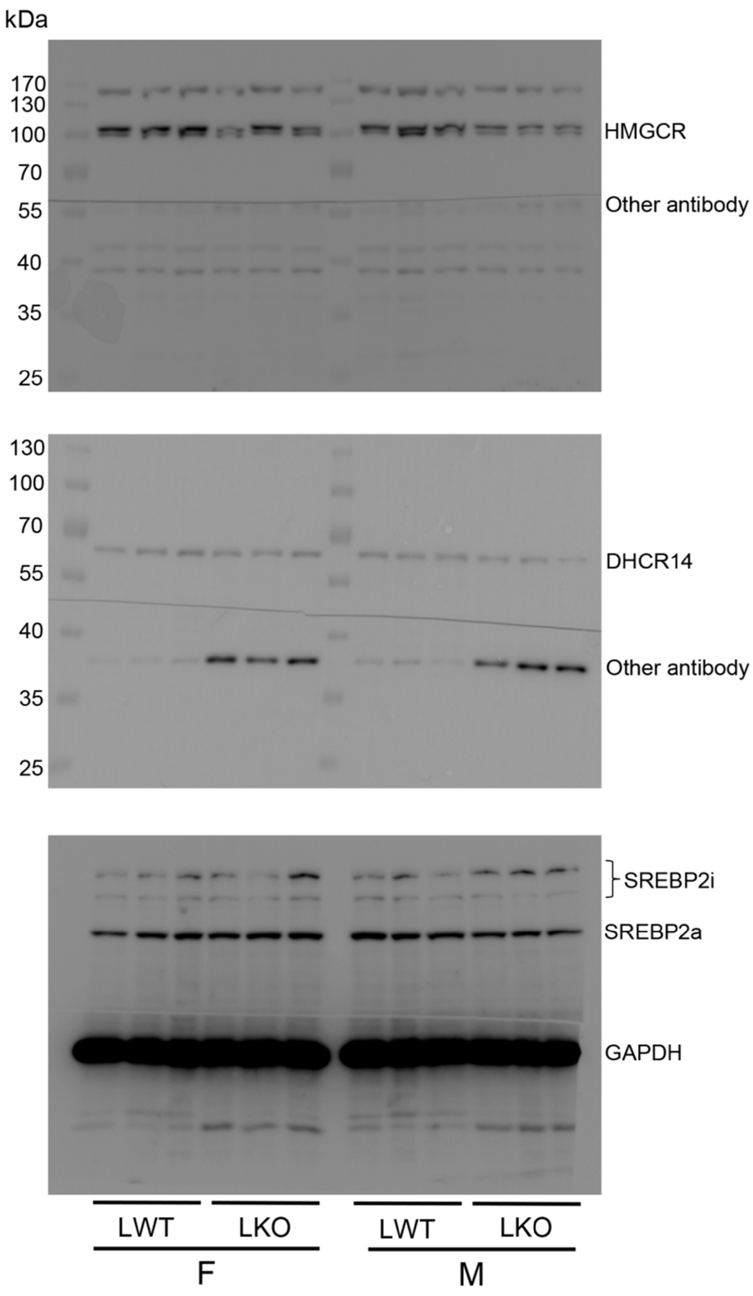

Supplementary Figure 10: Uncropped western blots of hepatic HMGCR, DHCR14 and SREBP2 of the LWT and LKO mice on the LFnC diet. The membranes were cut in order to stain each section with the respective antibody. GAPDH staining for HMGCR and DHCR14 blots is not shown. Western blot of SREBP2 was overexposed, thus enabling a proper quantification. SREBP2i – inactive membrane-bound form; SREBP2a – active nuclear form.

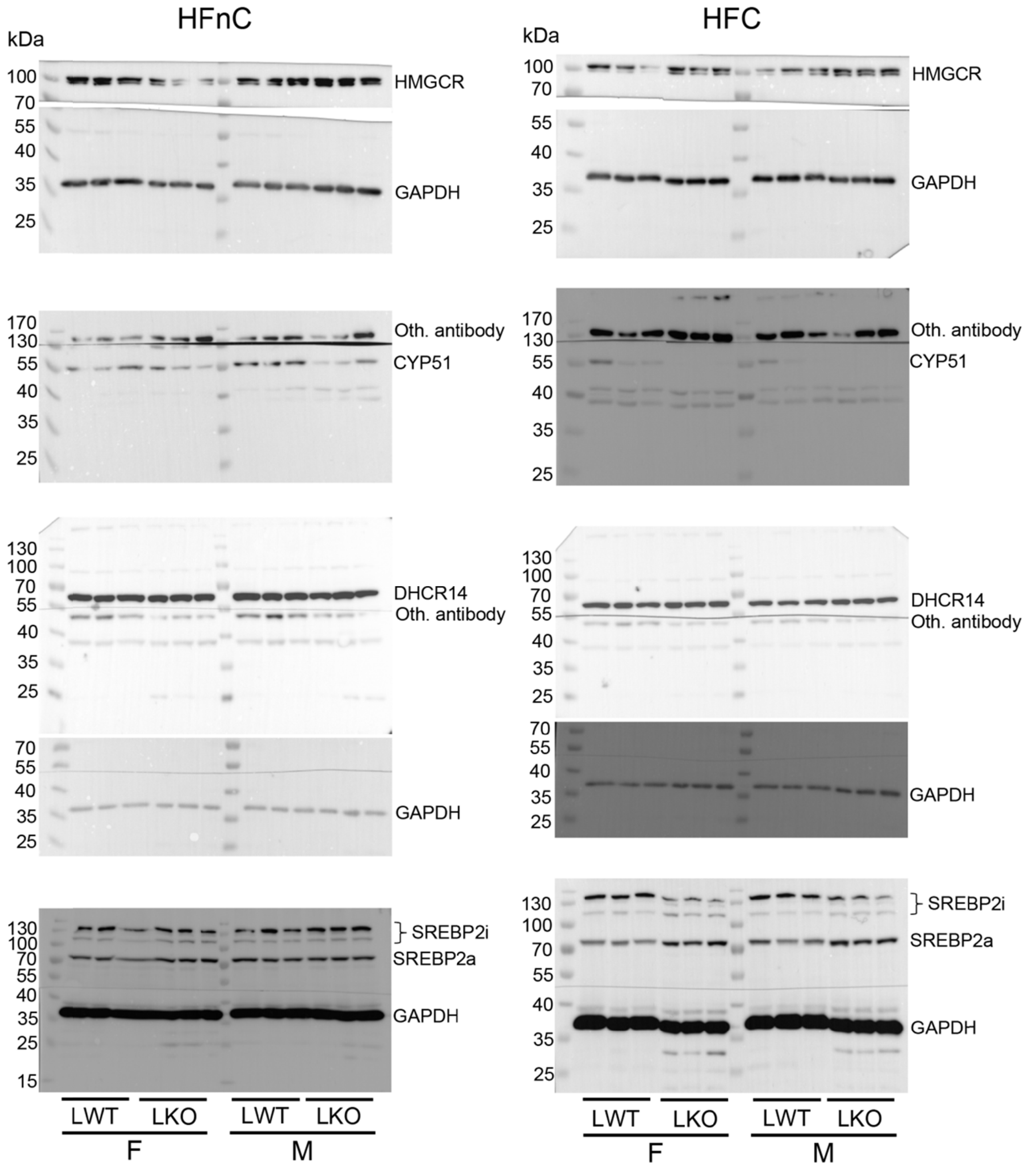

Supplementary Figure 11: Uncropped western blots of hepatic HMGCR, CYP51, DHCR14 and SREBP2 of the LWT and LKO mice on the HFnC and HFC diets. The membranes were cut in order to stain each section with the respective antibody. GAPDH staining for CYP51 is not shown. Western blot of SREBP2 was overexposed, thus enabling a proper quantification. SREBP2i – inactive membrane-bound form; SREBP2a – active nuclear form; HFnC, high-fat no-cholesterol diet; HFC, high-fat diet with cholesterol.

Supplementary Tables

Supplementary Table 1: Semi-quantitative histological evaluation of the liver H&E and Sirius red stained slides.

Supplementary Table 2: Differentially expressed genes for male LKO mice on LFnC diet.

Supplementary Table 3: Differentially expressed genes for female LKO mice on LFnC diet.

Supplementary Table 4: Enriched KEGG pathways according to pGSEA analysis in male LKOs on LFnC diet.

Supplementary Table 5: Enriched KEGG pathways according to pGSEA analysis in female LKOs on LFnC diet.

Supplementary Table 6: Enriched IPA canonical pathways for male LKOs on LFnC diet.

Supplementary Table 7: Enriched IPA canonical pathways for female LKOs on LFnC diet.

Supplementary Table 8: Plasma biochemistry of LWT and LKO mice of both sexes on high-fat diet without (HFnC) and with cholesterol (HFC).

Supplementary Table 9: Raw qPCR data for investigated genes involved in cholesterol synthesis and homeostasis.

Supplementary Table 10: Differentially expressed genes for male and female LKOs on HFnC diet.

Supplementary References

1 Keber, R. *et al.* Mouse knockout of the cholesterogenic cytochrome P450 lanosterol 14alpha-demethylase (Cyp51) resembles Antley-Bixler syndrome. *J Biol Chem* **286**, 29086-29097 (2011).

2 Andersen, H., Larsen, S., Spliid, H. & Christensen, N. D. Multivariate statistical analysis of organ weights in toxicity studies. *Toxicology* **136**, 67-77 (1999).

3 Geske, F. J. & Gerschenson, L. E. The biology of apoptosis. *Hum Pathol* **32**, 1029-1038 (2001).

4 van Diest, P. J. *et al.* Reproducibility of mitosis counting in 2,469 breast cancer specimens: results from the Multicenter Morphometric Mammary Carcinoma Project. *Hum Pathol* **23**, 603-607 (1992).

5 Lorbek, G., Perse, M., Horvat, S., Bjorkhem, I. & Rozman, D. Sex differences in the hepatic cholesterol sensing mechanisms in mice. *Molecules* **18**, 11067-11085 (2013).

6 Franceschini, A. *et al.* STRING v9.1: protein-protein interaction networks, with increased coverage and integration. *Nucleic Acids Res* **41**, D808-815 (2013).

7 Cline, M. S. *et al.* Integration of biological networks and gene expression data using Cytoscape. *Nat Protoc* **2**, 2366-2382 (2007).

8 In-Kwon, Y. & Johnson, R. A. A New Family of Power Transformations to Improve Normality or Symmetry. *Biometrika* **87**, 954-959 (2000).
